# Pan-Genomic Analysis Permits Differentiation of Virulent and Non-virulent Strains of *Xanthomonas arboricola* That Cohabit *Prunus* spp. and Elucidate Bacterial Virulence Factors

**DOI:** 10.3389/fmicb.2017.00573

**Published:** 2017-04-13

**Authors:** Jerson Garita-Cambronero, Ana Palacio-Bielsa, María M. López, Jaime Cubero

**Affiliations:** ^1^Departamento de Protección Vegetal, Instituto Nacional de Investigación y Tecnología Agraria y AlimentariaMadrid, Spain; ^2^Unidad de Sanidad Vegetal, Centro de Investigación y Tecnología Agroalimentaria de Aragón, Instituto Agroalimentario de Aragón, Universidad de ZaragozaZaragoza, Spain; ^3^Departamento de Bacteriología, Centro de Protección Vegetal y Biotecnología, Instituto Valenciano de Investigaciones AgrariasValencia, Spain

**Keywords:** stone fruit trees, almond, comparative genomics, bacterial spot disease

## Abstract

*Xanthomonas arboricola* is a plant-associated bacterial species that causes diseases on several plant hosts. One of the most virulent pathovars within this species is *X. arboricola* pv. *pruni* (*Xap*), the causal agent of bacterial spot disease of stone fruit trees and almond. Recently, a non-virulent *Xap*-look-a-like strain isolated from *Prunus* was characterized and its genome compared to pathogenic strains of *Xap*, revealing differences in the profile of virulence factors, such as the genes related to the type III secretion system (T3SS) and type III effectors (T3Es). The existence of this atypical strain arouses several questions associated with the abundance, the pathogenicity, and the evolutionary context of *X. arboricola* on *Prunus* hosts. After an initial characterization of a collection of *Xanthomonas* strains isolated from *Prunus* bacterial spot outbreaks in Spain during the past decade, six *Xap*-look-a-like strains, that did not clustered with the pathogenic strains of *Xap* according to a multi locus sequence analysis, were identified. Pathogenicity of these strains was analyzed and the genome sequences of two *Xap*-look-a-like strains, CITA 14 and CITA 124, non-virulent to *Prunus* spp., were obtained and compared to those available genomes of *X. arboricola* associated with this host plant. Differences were found among the genomes of the virulent and the *Prunus* non-virulent strains in several characters related to the pathogenesis process. Additionally, a pan-genomic analysis that included the available genomes of *X. arboricola*, revealed that the atypical strains associated with *Prunus* were related to a group of non-virulent or low virulent strains isolated from a wide host range. The repertoire of the genes related to T3SS and T3Es varied among the strains of this cluster and those strains related to the most virulent pathovars of the species, *corylina, juglandis*, and *pruni*. This variability provides information about the potential evolutionary process associated to the acquisition of pathogenicity and host specificity in *X. arboricola*. Finally, based in the genomic differences observed between the virulent and the non-virulent strains isolated from *Prunus*, a sensitive and specific real-time PCR protocol was designed to detect and identify *Xap* strains. This method avoids miss-identifications due to atypical strains of *X. arboricola* that can cohabit *Prunus*.

## Introduction

*Xanthomonas arboricola* species is traditionally conceived as a group of plant pathogenic bacteria associated with a wide range of host plants (Vauterin et al., 1995). Strains of this species have been classified into at least nine subinfraspecific groups or pathovars, which present a distinctive pathogenicity toward a delimited host range and conformed, in most of the cases, separate monophyletic groups (Fischer-Le Saux et al., 2015). Recently, the existence of non-virulent or saprophytic strains has been reported in plant hosts where pathogenic strains had been initially described (Essakhi et al., 2015; Jacques et al., 2016).

Within *X. arboricola*, pathovars *corylina, juglandis*, and *pruni*, which cause disease in nut, stone fruit trees and almond, have been considered as the most economically relevant groups since their first description in United States at the beginning of the 20th century (Boudon et al., 2005; Fischer-Le Saux et al., 2015). Symptoms caused by *X. arboricola* species are mainly described as blights as well as cankers and pustules on the aerial organs and tissues of the plant (Jacques et al., 2016). The negative effects in the crops are reflected in a yield reduction or in the inability to commercialize the damaged fruit (Lamichhane, 2014; Lamichhane and Varvaro, 2014). The appearance of several outbreaks of these pathovars which, in the case of the pathovars *corylina* and *pruni*, are regulated by quarantine policies in areas like the European Union (both pathogens are registered in the EPPO A2 list), and the possibility of future epidemics and spread of these pathogens to disease-free producing zones, have potentiated the efforts to understand the molecular diversity of the species (Anonymous, 2000; EFSA, 2014).

Very recent studies conducted by multilocus sequence typing and genome-wide based techniques, have provided a substantial increase in the knowledge associated with the genetic structure and diversity of *X. arboricola* strains (Essakhi et al., 2015; Fischer-Le Saux et al., 2015). These studies have revealed the existence of non-pathogenic or poorly virulent strains, isolated from at least seven plant genera, which composed a diverse phylogenetic group which is basal to the widespread epidemic groups of *X. arboricola*. The search of the type III secretion system (T3SS) and type III effectors (T3Es) in the different lineages of *X. arboricola*, based on a PCR analysis (Hajri et al., 2012; Essakhi et al., 2015), or by comparison with homologous sequences in the available genomes (Ibarra Caballero et al., 2013; Garita-Cambronero et al., 2014; Garita-Cambronero et al., 2016b; Cesbron et al., 2015; Higuera et al., 2015; Ignatov et al., 2015; Pereira et al., 2015; Harrison et al., 2016), have revealed a diverse gene profile of these components in *X. arboricola*; for instance, a large profile of T3Es for the pathovars *corylina, juglandis*, and *pruni* was determined in comparison with the other pathovars of the species (Hajri et al., 2012). In the same way, in some strains considered as non-pathogenic on walnut, the absence of a canonical T3SS or a variable low repertoire of T3Es was found. As occurred in other *Xanthomonas* species (White et al., 2009; Jacobs et al., 2015; Jacques et al., 2016), these significant genomic differences associated with virulence are interesting for evolutionary studies of the pathogenesis and the host specificity in *X. arboricola*.

Besides this, phylogenetic analysis based in the core genome sequence of *X. arboricola* (Cesbron et al., 2015; Garita-Cambronero et al., 2016a), revealed that three non-pathogenic strains isolated from walnut (*Juglans* sp.) and Santa Lucía SL-64 rootstock (*Prunus mahaleb*), did not group together with pathogenic strains isolated from these plant genera. Instead, these non-pathogenic strains were comprised in a group with several other low virulent strains, such as those of the pathovar *celebensis*, a pathogen of banana (*Musa* spp.) (Harrison et al., 2016), or with the strain 3004 of *X. arboricola* isolated from barley (*Hordeum vulgare*) (Ignatov et al., 2015).

The existence of non-pathogenic strains has aroused the concern on how abundant are they in plants, and the possibility of their misidentification as pathogenic strains by the current diagnostic approaches. Moreover, they could be in useful to obtain some clues related to the evolution of pathogenesis in *X. arboricola*. Recalling all these recent advances, our goal was to deepen the characterization of the genomic features of three atypical strains isolated from *Prunus* spp., in order to determine how these key features associated with pathogenesis varied among atypical and pathogenic strains of *X. arboricola* pv. *pruni*, as well as to determine if these variants could be used to design precise molecular tools to differentiate these two groups when they cohabit the same *Prunus* host.

## Materials and methods

### Bacterial strains and classification using multilocus sequence analysis

Thirty-one previously characterized strains of *X. arboricola* (Young et al., 2008; Palacio-Bielsa et al., 2011; Pothier et al., 2011b; Garita-Cambronero et al., 2016a; López-Soriano et al., 2016) from the pathovars *pruni, corylina, juglandis*, and *populi* were utilized. Besides, 40 strains showing *Xanthomonas*-like colonies were collected during the Spanish outbreaks of bacterial spot disease of stone fruit trees and almond, as well as from routine screenings performed on Spanish nurseries (Table S1). These strains were screened for identification as *Xanthomonas arboricola* pv. *pruni* (*Xap*). All the bacterial strains listed in Table S1 are available in the collections from the Instituto Valenciano de Investigaciones Agrarias (IVIA, Valencia, Spain) and the Centro de Investigación y Tecnología Agroalimentaria de Aragón (CITA, Aragón, Spain).

Bacterial strains were cultured on Luria Bertani (LB) 1.5% agar plates or in LB broth at 27°C for 48 h. The commensal bacterial strains, isolated from *Prunus* and used in this study (Table S1), were identified to genus level based on the partial sequence of the 16S rDNA gene according to a method described previously (Lagacé et al., 2004).

For an initial *Xap* classification, a real-time PCR reaction in the gene *ftsX* of an ABC transporter (Palacio-Bielsa et al., 2011, 2015), and a multiplex PCR for plasmid pXap41 (Pothier et al., 2011b) were performed. Those strains that showed a positive result only for the real-time assay were considered as *Xap*-look-a-like strains, and were further identified according to a multilocus sequence typing scheme (MLSA) based in the partial sequences of the housekeeping genes *dnaK, fyuA, gyrB* and *rpoD* (Young et al., 2008).

Additionally, sequences of these housekeeping genes from the *Prunus*-non-virulent *X. arboricola* strain CITA 44 and sequences from *X. arboricola* pathovars *celebensis* (CFBP 3523 = ICMP 1488 = NCPPB 1832), *corylina* (CFBP 1159 = ICMP 5726 and CFBP 1846), *juglandis* (CFBP 2528 = ICMP 35 and IVIA 2113), *populi* (CFBP 3123) and *pruni* (CFBP 2535 = ICMP 51, CFBP 5530, Xap 33 = CITA 33 and IVIA 2626.1), as well as *X. citri* subsp. *citri* strain CFBP 2525 = ICMP 24, included as outgroup, were obtained from the National Center for Biotechnology Information database (NCBI).

Purified PCR products were sequenced at STAB VIDA (Lisbon, Portugal), and edited using Geneious (Kearse et al., 2012). Obtained nucleotide sequences were aligned with ClustalW version 1.83 (Hall, 2011) using default parameters. Both ends of each alignment were trimmed to the following sizes: *dnaK*, 842 positions; *fyuA*, 601 positions; *gyrB*, 631 positions and *rpoD*, 759 positions. Then, they were aligned and concatenated to give a total length of 2,833 nucleotide positions. For the analysis of the concatenated gene dataset, Tamura-Nei (TN93) model was selected and maximum likelihood trees, using 1,000 bootstrap re-samplings, were generated using MEGA 6.0 software (Tamura et al., 2013).

Nucleotide sequences were deposited in GenBank. Accession numbers for the partial sequences of the genes used in this study are: KX357115 to KX357120 for *dnaK*; KX357133 to 357138 for *fyuA*; KX357127 to KX357132 for *gyrB* and KX357121 to KX357126 for *rpoD*.

### Study of the type III secretion system and type III secreted proteins gene repertory

Six strains isolated from *Prunus* spp. and classified as *Xap*-look-a-like, as well as the pathogenic *Xap* strains CITA 33 and CFBP 5530, and the *Prunus*-non-virulent strain CITA 44, were typed by PCR for 11 genes related to structural and regulatory components of the T3SS, 19 genes for the T3Es and two genes that encoded the type III secreted proteins (T3SPs) *hpaA* and *hrpW* predicted in *Xap* (Hajri et al., 2012; Garita-Cambronero et al., 2016a). PCR reactions were performed according to the conditions proposed previously (Hajri et al., 2012) with the exception of the T3SS genes, *hrpD5* and *hrpF*, as well as the T3SP *hpaA* and the T3Es genes *xopAQ* and *xopZ*, for which new sets of primers were designed based in orthologues available in databases for *X. arboricola* (Table 1). PCR amplifications with the primers for *hrpD5, hrpF, hpaA, xopAQ*, and *xopZ* were performed in 20 μl of PCR reaction containing 1X PCR buffer (10 mM Tris-HCl, 50 mM KCl, 0.1% Triton X-100 [pH 9.0]); 0.5 μM of each primer; 0.25 U *Taq* DNA polymerase (Biotools, Madrid, Spain); 0.2 mM each dNTP (Biotools Madrid, Spain); 1.5 mM MgCl_2_ and 1.0 μg/μl of DNA template. PCR conditions consisted in an initial denaturation step of 94°C for 2 min, 30 cycles of denaturation at 94°C for 1 min, annealing at 60°C for 1 min, extension at 72°C for 2 min and a final extension step at 72°C for 10 min. PCR products were visualized in 2% agarose gel containing Midori Green nucleic acid gel staining solution (Nippon Genetics Europe, Dueren, Germany).

**Table 1:** **PCR primers used to amplify a partial region of some genes associated with type III secretion system (T3SS), type III effectors (T3E) and other type III secreted proteins (T3SPs) in *X. arboricola***.

**T3SS/T3E gene**	**Forward primer**	**Reverse primer**	**Fragment size (pb)**
*hpaA*	ATGATCCGGCGCATTTCG	GCGATGCTGACCCGGC	269
*hrpD5*	ATCGAGGTGGATGCAGATGG	CGGCAGGGAAGTCAGGTG	795
hrpF^*^	TCTACCTCTGACGGATGACG	GTCGCCCTGCGAGCC	516
*hrpF*^¥^	TCTACCTCTGACGGATGACG	GGTCGGCAAAGTCGTAGAGG	947
*xopAQ*	ATCGGGAGACACAGGGTGTA	CTTCTGAGGTAGCGGAC	146
*xopZ*	CATTCGTCGCGGATCAACAC	GAAAGCCGGGAAGGATGTCT	196

* *Primers used to amplify the ortholog of hrpF in pathogenic strains of X. arboricola pathovars corylina, juglandis and pruni*.

¥ *Primers used to amplify the ortholog of hrpF in X. arboricola pv. celebensis and non-pathogenic strains of X. arboricola*.

### Pathogenicity tests

Pathogenicity tests on barley (*H. vulgare*), *Nicotiana benthamiana, Nicotiana tabacum, Prunus persica* (rootstock GF-305) and tomato (*Solanum lycopersicum*) were carried out for the six *Xap*-look-a-like strains as well as for the pathogenic strain of *Xap* CITA 33 and the non-virulent strain of *X. arboricola* CITA 44. Bacterial suspensions in sterile phosphate buffered saline (PBS pH = 7.5), adjusted to a final concentration of 1 × 10^6^ colony forming units (CFU)/ml, were infiltrated in three leaves per plant using a syringe without needle and sterile PBS was utilized as blank control. All the infiltrated plants were kept in a growth chamber with high humidity and 16 h of light and 8 h of darkness at 26 and 22°C, respectively. Infiltration results were graphically recorded at 0, 7, 14, and 21 days post inoculation (dpi). After 21 dpi, the infiltrated leaves were macerated on sterile distilled water and tenfold dilution of the macerated were plated on YPGA (0.5% yeast extract, 0.5% bactopeptone, 1.0% glucose, 2.0% agar) supplemented with 250 mg/l of cycloheximide. Colonies showing a *Xanthomonas*-like phenotype were confirmed as *Xap*-look-a-like using a real time PCR protocol indicated above (Palacio-Bielsa et al., 2011, 2015). Additionally, for *P. persica* rootstock GF-305, colonies were counted in order to determine the bacterial concentration in the inoculated tissue at the end of the assay (Ah-You et al., 2007).

### Genome sequencing and comparison

**Figure 1:**
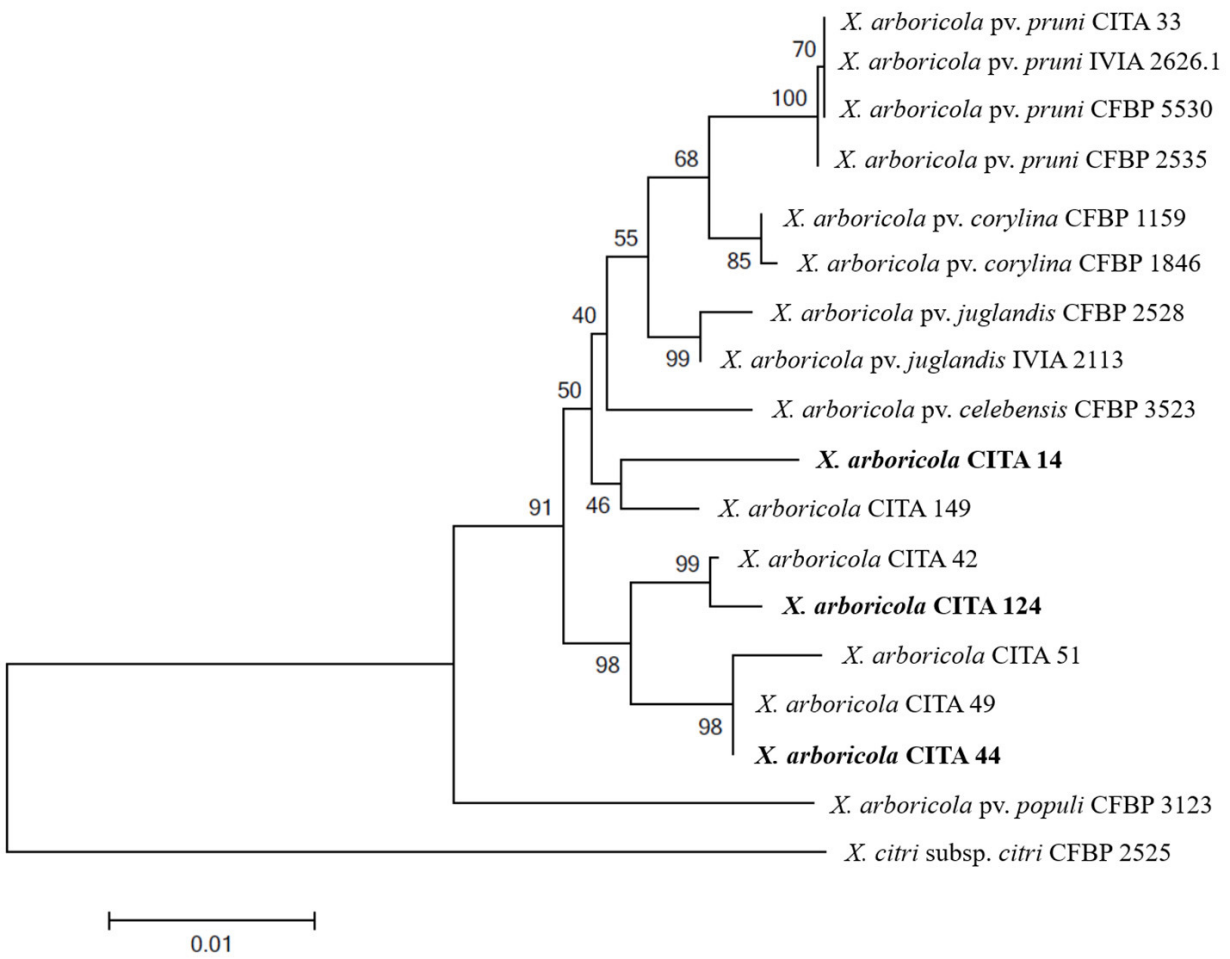
**Maximum likelihood tree of concatenated sequences of the genes *dnaK*, *fyuA*, *gyrB* and *rpoD* of non-virulent *Xanthomonas arboricola* strains isolated from *Prunus* spp**. For comparative purposes pathogenic strains of *X. arboricola* pv. *pruni* isolated from *Prunus* spp. and *X. arboricola* strains isolated from other hosts were included. *X. citri* subsp. *citri* was used as an outgroup. Bootstrap values of 1,000 replicates are represented over or below the branches. Selected strains for subsequent whole genome sequencing are in bold.

From the six *Xap*-look-a-like strains analyzed previously, one representative of each cluster, according to the MLSA analysis (Table S1; Figure 1), was selected for genome sequencing. Genome sequencing conditions and features for CITA 44 have been discussed in a previous paper (Garita-Cambronero et al., 2016b). In addition, in this study the genome features of the *Xap*-look-a-like strains CITA 14 and CITA 124 are described. For these two strains, the genome sequencing and assembly conditions have been previously announced and deposited at DDBJ, EMBL, GenBank databases under the accession numbers LXIB00000000 for CITA 14 and LXKK00000000 for CITA 124 (Garita-Cambronero et al., 2016c).

The assembled draft genome sequence of strains CITA 14 and CITA 124 were automatically annotated using the NCBI's prokaryotic annotation pipeline (Tatusova et al., 2013). Signal peptides and transmembrane domains were predicted using the signalP 4.1 (Petersen et al., 2011) and the TMHMM 2.0 (Krogh et al., 2001) servers. The assignment of genes to each cluster orthologous group (COG) and its Pfam domain was performed with the NCBI's conserved domain database using an expected value threshold of 0.001 (Marchler-Bauer et al., 2014). The circular genome maps of the draft genome sequences of *X. arboricola* strains CITA 14 and CITA 124, representing the COG categories of the genes, were constructed using CGView (Stothard and Wishart, 2005). The contigs of both strains were arranged by Mauve (Darling et al., 2004, 2010) using the complete genome sequence of *X. arboricola* pv. *juglandis* Xaj417 as the reference (Pereira et al., 2015).

The genome sequence variation among CITA 14, CITA 124 and the publicly available genomes of *X. arboricola* (strains 3004, CFBP 7634, CFBP 7651, and CITA 44; Cesbron et al., 2015; Ignatov et al., 2015; Garita-Cambronero et al., 2016b), *X. arboricola* pv. *celebensis* (NCPPB 1630 and NCPPB 1832; Harrison et al., 2016), *X. arboricola* pv. *corylina* (NCCB 100457; Ibarra Caballero et al., 2013), *X. arboricola* pv. *juglandis* (CFBP 2528, CFBP 7179, Xaj2, and Xaj417; Cesbron et al., 2015; Higuera et al., 2015; Pereira et al., 2015) and *X. arboricola* pv. *pruni* (CITA 33, IVIA 2626.1, MAFF 301420, and MAFF 301427; Garita-Cambronero et al., 2014; Garita-Cambronero et al., 2016b) (Table S2), were determined by using sequence-based and sequence content approaches (Snipen and Ussery, 2010).

Comparison among genome sequences based on sequence alignment and evolutionary analysis, of the shared protein coding sequences (CDS), were determined using Roary (Page et al., 2015). Contigs of the 17 genome sequences of *X. arboricola* were ordered by Mauve. Afterwards, all the genome sequences were automatically annotated using PROKKA (Seemann, 2014) and used as the input for the search of shared homologous genes among the studied strains.

Those CDS shared by all the analyzed genomes of *X. arboricola*, with an identity and a coverage percentage over 80%, were considered as homologous genes. The concatenated sequences of the genes that composed the core genome sequence of *X. arboricola* were aligned using the PRANK (Löytynoja and Goldman, 2008) and subsequently, a maximum likelihood tree (1,000 bootstrap resamplings) was constructed to determine the phylogenetic position of the strains CITA 14 and CITA 124 within *X. arboricola*. Maximum likelihood tree was performed with RaxML (Stamatakis, 2014) and visualized using Dendroscope (Huson et al., 2007).

Additionally, the gene-content comparison was performed using the R implemented package for microbial-pangenomics micropan (Snipen and Liland, 2015). The CDS were obtained from nucleotide genome sequences of the 17 strains mentioned above using Prodigal v2.6.1 (Hyatt et al., 2010). To determine the similarity of the proteins within and across the genomes, a reciprocal all-against-all BLAST search was performed using the blastAll package. BLAST distance between sequences was obtained using the bDist package. A hierarchical clustering was performed using bClust, and a complete linkage function was selected with a liberal threshold of 0.80. A similarity matrix, with the number of protein sequences contained in each cluster for each genome, was constructed using the panMatrix function and Jaccard distance was calculated. The matrix was used to perform a principal component analysis for showing how the genomes were distributed in the space according to the two first principal components which revealed the dominant differences between them, and this was computed using the panpca and plotScores functions. Additionally, the similarity of the analyzed genomes was represented in a pan-genome tree using the panTree function (Snipen and Ussery, 2010). Tree construction was based in the distance between genomes according to the Manhattan distance. Bootstrap values were calculated by re-sampling the columns of the similarity matrix and the re-clustering of these data, therefore, the bootstrap value represented was the percentage of the re-sampled trees that showed a similar node.

### Genes associated with pathogenicity in *X. arboricola* strains isolated from *Prunus* spp.

In order to determine potential groups of genes with a putative function related to pathogenesis, genes with an identity and a coverage percentage over 80% associated with tonB-dependent transporters (TBDTs), sensors of the two-component regulatory system (STCRs), methyl accepting chemotaxis proteins (MCPs), flagella, type IV pilus, non-fimbrilar adhesins, production of xanthan, quorum-sensing regulation, repertoire of cell-wall degrading enzymes, type II, III and IV secretion systems, as well as T3Es and T3SPs, previously reported in *Xanthomonas* spp. (da Silva et al., 2002; Filloux, 2004; Chevance and Hughes, 2008; He and Zhang, 2008; Vorhölter et al., 2008; Wang et al., 2008; White et al., 2009; Subramoni et al., 2010; Szczesny et al., 2010; Guo et al., 2011; Mhedbi-Hajri et al., 2011; Potnis et al., 2011; Ryan et al., 2011; Hajri et al., 2012; Vandroemme et al., 2013; Guglielmini et al., 2014; Li et al., 2014; Cesbron et al., 2015; Dunger et al., 2016; Nascimento et al., 2016), were searched in the genomes.

The presence of the putatively virulence-associated plasmid pXap41 (Pothier et al., 2011b) was also evaluated in the analyzed genomes based in the nucleotide sequence similarity, graphically represented using the BLAST Ring Image Generator (BRIG) tool (Alikhan et al., 2011) and blastn was used for the sequence comparative analysis with an expected value threshold of 0.001.

### A molecular tool to differentiate *X. arboricola* pv. *pruni* from atypical strains of *X. arboricola* associated with *Prunus* spp.

In order to discriminate *Xap* from other atypical *X. arboricola* strains present in *Prunus* spp., a partial sequence of the *xopE3* gene that is encoded on the pXap41 plasmid, described as specific for *Xap* (Pothier et al., 2011b), was used. Sequences of the *xopE3* gene available in GenBank database from strains CITA 33 (GenBank locus tag DK27_00095), IVIA 2626.1 (AN652_04270), MAFF 301420 (XPR_2580), MAFF 301427 (XPN_1257) and CFBP 5530 (XAP_pXAP410005) were aligned with ClustalW and the consensus sequence used as template for *xopE3* primers and probe design using the ABI PRISM Primer Express software v. 2 (Applied Biosystems, Foster City, CA). Specificity of the primers was firstly evaluated *in silico* using the Primer-BLAST tool available at NCBI. Graphical representation of the primers and the probe hybridization was performed in a set of *X. arboricola* genome sequences using the BRIG software.

Real-time PCR was conducted in a total volume of 25 μl containing, 12.5 μl of GoTaq probe qPCR MasterMix (Promega, Madison, WI, USA), 0.4 μM of each primer, 150 nM of TaqMan probe, and 5 μl of sample. Real-time PCR amplifications were performed in an ABI 7,500 Fast thermocycler (Applied Biosystems, Foster city, CA) and consisted of an initial denaturation step of 95°C for 5 min followed by 45 cycles, each one of 1 min at 95°C and 1 min at 59°C.

### Specificity of the real-time PCR test

Specificity of the real-time PCR test was assessed in 99 bacterial strains, which comprised 54 strains of *Xap*, seven strains of *Xap*-look-a-like, ten strains from other pathovars of *X. arboricola*, 11 strains from other species of *Xanthomonas*, eight strains from other genera of phytopathogenic bacteria, and nine strains from the natural microbiota of *Prunus* spp. (Table S1). Bacterial suspensions of 10^8^ CFU/ml were treated at 95°C during 10 min and used for real-time PCR reactions. Sterile distilled water was used as negative control. Additionally, a real-time PCR protocol previously described (Palacio-Bielsa et al., 2011, 2015) was also applied on all the 99 bacterial strains tested for *xopE3* gene.

### Sensitivity of the real-time PCR test

Serial dilutions from 10 to 10^8^ CFU/ml of a 48 h LB broth culture of strain CITA 33 were prepared in sterile distilled water and heat-treated (95°C for 10 min) for real-time PCR reactions. Additionally, serial dilutions of pure bacterial DNA (QIamp DNA miniKit, Qiagen, Hilden, Germany), ranging from 0.001 pg/μl to 10^8^ pg/μl were also prepared in sterile distilled water. A volume of 5 μl of each dilution was used as template for amplification. Seven replicates of each sample were evaluated in each experiment, and the experiment was repeated in three independent assays. Appropriate negative controls containing no bacteria or no DNA were subjected to the same procedure. The limit of detection of the test was defined as the lowest target amount giving positive results in at least 15 of the 21 total reactions tested in the three independent assays (Caraguel et al., 2011). Analysis of variance was used to test for differences in the threshold cycles (C_ts_) at each bacterial concentration in the three independent assays. Statistical analyses were performed by using Statgraphics Plus v.5.1 software. The amplification efficiency of the protocol for each kind of sample was calculated as described previously (Palacio-Bielsa et al., 2011, 2015). Linear regression curves representing the C_ts_ of each reaction were plotted against the logarithmic values of bacterial or DNA concentration. The slope of the curves (*k*) was used to determine the amplification efficiency (*E*) according to the equation *E* = 10^[−1/*k*]^, where *E* = 2 corresponded to 100% efficiency.

## Results

### Characterization of atypical strains of *X. arboricola* associated with *Prunus* spp.

A total of 40 strains isolated from *Prunus* spp. and phenotypically similar to *X. arboricola* were initially identified as *Xap* by using a real-time PCR protocol (Palacio-Bielsa et al., 2011, 2015). Additionally, the plasmid pXap41, which is considered a specific feature of *Xap*, was not detected by PCR amplification of the genes *repA1, repA2* and *mobC* (Pothier et al., 2011b) from strains CITA 14, CITA 42, CITA 49, CITA 51, CITA 124, and CITA 149 and, therefore, they were not considered as *Xap* but *Xap*-look-a-like strains.

In order to further characterize, six *Xap*-look-a-like strains mentioned above, and the *Prunus*-non-virulent strain CITA 44 (Garita-Cambronero et al., 2016b), were analyzed using a MLSA scheme based in partial sequences of the genes *dnaK, fyuA, gyrB* and *rpoD* (Young et al., 2008). The Maximum likelihood analysis of the concatenated sequences revealed that none of the *Xap*-look-a-like strains could be consistently clustered with any of the reference strains that belong to the pathovars described within *X. arboricola*. On the contrary, these strains were distributed in three separated clusters, one composed by strains CITA 14 and CITA 149, another composed by strains CITA 44, CITA 49 and CITA 51 and a third one composed by strains CITA 42 and CITA 124. These clusters were located in a basal phylogenetic position with respect to most of the strains used as reference. Sequence analysis of the concatenated sequence (2,833 nucleotide positions) revealed a mean similarity of 98.30 ± 0.2% between the *Xap*-look-a-like strains and the remaining ten strains of *X. arboricola* (Figure 1). As expected, according to other studies (Essakhi et al., 2015; Fischer-Le Saux et al., 2015), the phylogenetic clustering deduced from individual genes did not result in the same phylogenetic arrangement observed, which reinforced the need of a compendium of genetic characters as used in the MLSA (Figure S1).

### T3SS, T3SPs and T3Es repertoire in *Xap*-look-a-like strains

Conventional PCR typing of 32 genetic determinants of the T3SS, its related T3SPs and T3Es, brought out a variable repertoire in the six *Xap*-look-a-like strains. The structural and regulatory components of the T3SS were only detected in strains CITA 14 and CITA 149, which harbored some of the 11 components tested. Similarly, only strains CITA 14 and CITA 149 harbored five and two T3SPs and T3Es, respectively. On the other hand, CITA 42, CITA 49, CITA 51 and CITA 124 did not harbor any of these genes. As expected, *Xap* strains CITA 33 and CFBP 5530, showed positive amplification from all the analyzed genes, while the non-virulent strain CITA 44 resulted negative (Table 2).

**Table 2:** **Components of the type three secretion system, repertoire of the type three effectors and other secreted proteins, presence of the plasmid pXap41 and pathogenicity of *X. arboricola* strains isolated from *Prunus* spp**.

	**Gene/Strains**	**CITA 14**	**CITA 42**	**CITA 44**	**CITA 49**	**CITA 51**	**CITA 124**	**CITA 149**	**CFBP 5530^P^**	**CITA 33^P^**
Components of the type III secretion system	*hrcC*									
	*hrcJ*									
	*hrcN*									
	*hrcR*									
	*hrcS*									
	*hrcT*									
	*hrcU*									
	*hrcV*									
	*hrpB1*									
	*hrpD5*									
	*hrpF*									
Type III effectors and other type III secreted proteins	*avrBs2*									
	*avrXccA2*									
	*hpaA*									
	*hrpW*									
	*xopA*									
	*xopAF*									
	*xopAH*									
	*xopAI*									
	*XopAQ*									
	*xopE2*									
	*xopE3*									
	*xopF1*									
	*xopG*									
	*xopK*									
	*xopL*									
	*xopN*									
	*xopQ*									
	*xopR*									
	*xopV*									
	*xopX*									
	*xopZ*									
pXap41	*repA1*									
	*repA2*									
	*mobC*									
Pathogenicity	*Hordeum vulgare*	N, C	NS	NS	NS	NS	NS	NS	ND	NS
	*Nicotiana benthamiana*	N, C	C	C	MC	C	MC	N, C	ND	N, C
	*N. tabacum*	N	C	C	C	C	C	C	ND	N
	*Solanum lycopersicum*	N	MN, C	NS, MC	N, C	N, C	N, C	N, C	ND	N, C
	*Prunus persica* (GF-305)	MN	N	NS	N	N	MN	N	ND	N, C
	CFU/ml 21 dpi	0–10^5^	10^1^–10^5^	10^2^–10^4^	10^2^–10^4^	10^3^–10^5^	10^3^–10^5^	10^3^–10^5^	ND	10^6^–10^10^
	Real-time PCR^*^	+	+	+	+	+	+	+	ND	+

*Positive/negative PCR amplification are represented in gray or white, respectively. P, Prunus pathogenic strains of X. arboricola pv. pruni (Xap), the remaining tested strains were considered as atypical Xap-look-a-like strains; N, necrosis; C, chlorosis; NS, not visible symptoms; MC, mild chlorosis; MN, mild necrosis; ND, no data*.

* *According to the protocol described by Palacio-Bielsa et al. (2011, 2015)*.

*CITA, Centro de Investigación y Tecnología Agroalimentaria de Aragón, Zaragoza, Spain; CFBP, Collection Française de Bactéries Phytopathogénes, Angers, France*.

### Pathogenicity of the *Xap*-look-a-like strains

In addition to the variability found in the T3SS components, its related effectors and other secreted proteins, the ability of the *Xap*-look-a-like strains to cause disease symptoms after bacterial infiltration on leaves of barley, *N. benthamiana, N. tabacum*, tomato and the susceptible peach rootstock GF-305 was evaluated. None of the assayed strains were able to cause disease symptoms on barley, with the exception of strain CITA 14, which caused necrosis and chlorosis in the infiltrated zone. Strains CITA 14, CITA 149, and *Xap* strain CITA 33 were able to cause necrosis and chlorosis in *N. benthamiana* and *N. tabacum*. On the contrary, strains CITA 42, CITA 49, CITA 51, CITA 124 and the *Prunus*-non-virulent strain CITA 44 only showed a chlorosis effect after 21 dpi. In tomato at 21 dpi, all the assayed strains, with the exception of CITA 44, were able to cause necrosis and, in most of the cases, the necrotic area was surrounded by a chlorotic halo. Infiltration on peach rootstock GF-305 showed that only CITA 44 did not cause damage on the leaves; however, the remaining strains CITA 14, CITA 42, CITA 49, CITA 51, CITA 124 and CITA 149 caused necrosis in the infiltrated area after 7 dpi, but these necrotic spots did not expand beyond this zone and were different to typical bacterial spot symptoms.

On the other hand, the *Xap* strain CITA 33, caused necrosis in the infiltrated area and these necrotic zones were surrounded by a chlorotic halo (Figure S2). In addition, in GF-305, variation in bacterial populations on the infiltrated leaves was determined after 21 dpi, and all the *Xap*-look-a-like strains, as well as strain CITA 44, showed a reduction in their bacterial populations leading to concentrations equal or lower than 10^5^ CFU/ml. For strain CITA 33, the bacterial concentration increased from 10^6^ to 10^10^ CFU/ml by the end of the assay (Table 2). Positive results in real-time PCR analysis of the isolated colonies after 21 dpi, using the standardized protocol for *Xap* detection (Palacio-Bielsa et al., 2011, 2015), corroborated that the re-isolated strains corresponded to the same inoculated at the beginning of the study.

### General features of whole genomes of *X. arboricola* strains CITA 14 and CITA 124

According to the results obtained in the MLSA analysis, one member of each one of the three clusters (CITA 14, CITA 44 and CITA124) observed for the *Xap*-look-a-like strains was selected for whole genome sequencing (Figure 1). The draft genome sequence of the *Prunus*-non-virulent strain CITA 44 has been reported and analyzed previously (Garita-Cambronero et al., 2016a). Moreover, main sequencing and structural features of CITA 14 and CITA 124 genome sequences have been previously announced (Garita-Cambronero et al., 2016c).

**Figure 2:**
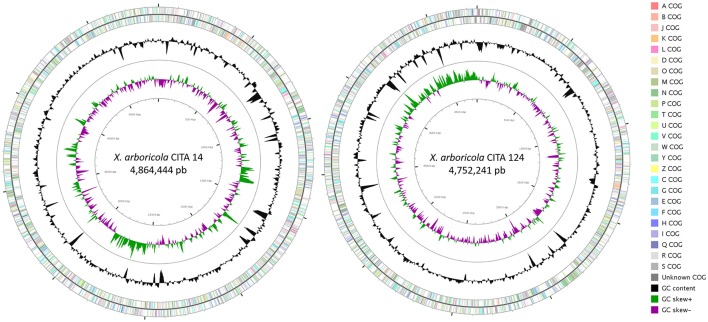
**Graphical circular representation of the draft genome of the *Prunus*-non-virulent strains of *Xanthomonas arboricola* CITA 14 and CITA 124**. The contigs were arranged by Mauve, using the genome sequence of *X. arboricola* pv. *juglandis* strain Xaj417 as reference. COG categories were assigned to predicted genes using the NCBI's conserved domain database. Circular map was constructed using CGview. From outside to center: Genes on forward strand; genes on reverse strand; GC content; GC skew.

Draft genome sequence of CITA 14 was 4,864,444 bp in length with an average GC content of 65.60%; 3,870 over 3,974 genes predicted were identified as protein coding genes and a putative function was assigned to 2,991 of them (Table 3). This strain presented four rRNAs and 53 tRNAs. In the case of CITA 124, its draft genome sequence was 4,752,241 bp in length with an average GC content of 65.80%. A total of 4,004 genes were predicted and, among them, 3,798 were identified as protein coding genes with a putative function assigned to 2,838 of them. In addition, three rRNA and 50 tRNA genes were predicted (Table 3). From the total of genes with a predicted function, a COG functional category was assigned to 3,090 and 2,668 genes in CITA 14 and CITA 124, respectively. Those genes related to the amino acid and carbohydrate transport and metabolism, as well as those associated with translation and ribosomal structure and biogenesis, were predominant for both strains (Figure 2). Moreover, CITA 14 and CITA 124 presented 3,320 and 2,668 genes, respectively, with a protein domain in the Pfam database. Finally, a total of 625 and 942 genes of CITA 14 presented peptide cleavage signals and transmembrane helices, respectively. While, in CITA 124, 569 genes with peptide cleavage sites and 954 genes with transmembrane helices were predicted (Table 3, Table S3).

**Table 3:** **Genome sequence information and statistics of the atypical strains of *X. arboricola* strains CITA 14 and CITA 124, isolated from *Prunus***.

**Property/Attribute**	**CITA 14**	**CITA 124**
	**Value**	**Value**
Sequencing platform	Ion Torrent PGM	Ion Torrent PGM
Fold coverage	100x	50x
Assemblers	CLC and MIRA 4.0	CLC and MIRA 4.0
Genome annotation	NCBI-PGAP	NCBI-PGAP
Locus tag	A7D01	A7D35
Genbank ID	LXIB00000000	LXKK00000000
Genome size (bp)	4,864,444	4,752,241
DNA G+C (%)	65.60	65.80
Total genes	4,061	4,086
Protein coding genes	3,870	3,798
RNA genes	87	82
Pseudo genes	104	206
Genes with function prediction	2,991	2,838
Genes assigned to COGs	3,090	2,668
Genes with Pfam domains	3,320	2,668
Genes with signal peptides	625	569
Genes with transmembrane helices	942	954
CRISPR repeat unit	1	0

*CITA, Centro de Investigación y Tecnología Agroalimentaria de Aragón, Zaragoza, Spain*.

**Figure 3:**
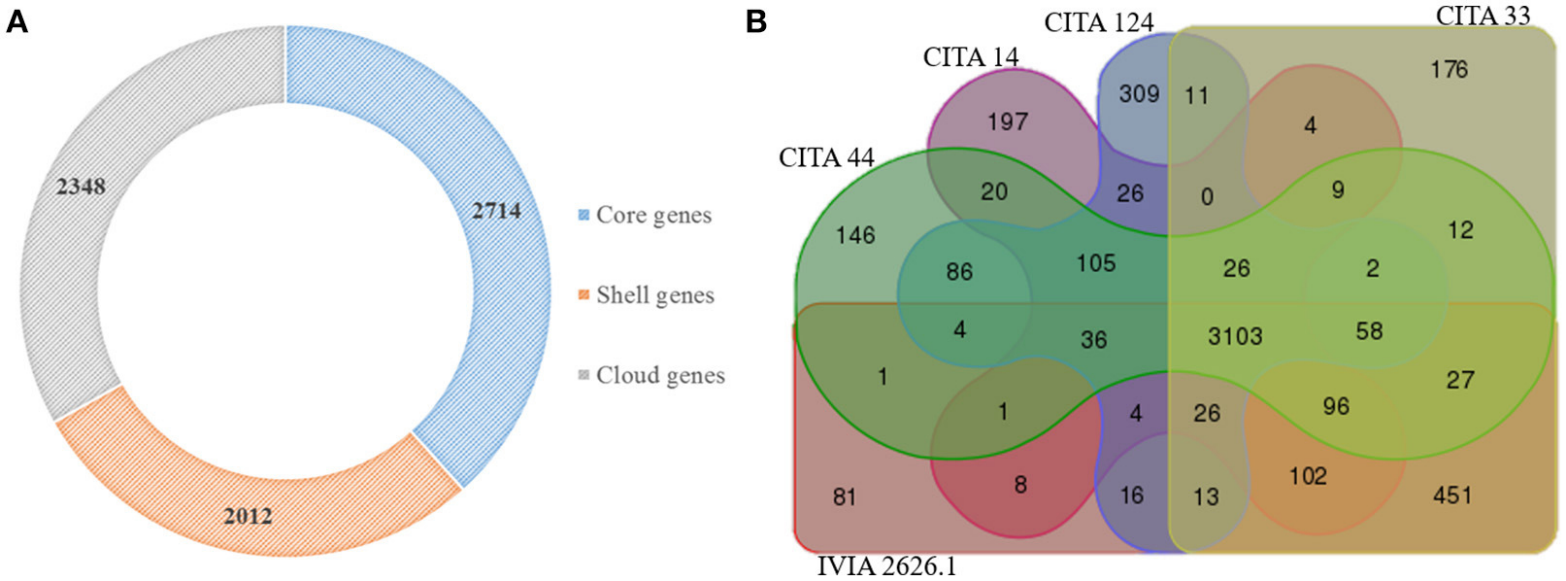
**Potential groups of orthologous genes present in *Xanthomonas arboricola***. Core, shell and cloud groups of orthologous genes shared by 17 genome sequences of *X. arboricola* **(A)**. Venn diagram showing the groups of orthologous genes shared by five genome sequences of pathogenic (CITA 33 and IVIA 2626.1) and non-virulent (CITA 14, CITA 44, and CITA 124) strains of *X. arboricola* isolated from *Prunus* spp. **(B)**.

A comparative gene content analysis was performed among the genome sequences of the two *Prunus*-non-virulent *Xap*-look-a-like strains, CITA 14 and CITA 124, and 15 genome sequences of other strains of *X. arboricola*. As result, a total of 7,074 potential groups of homologous genes were found in the 17 analyzed genomes, from which 2,714 were shared by all the *X. arboricola* strains and comprised the core group of orthologus genes. CITA 14 and CITA 124 presented 76 and 124 unique cluster genes, respectively, which distinguished them from the other 15 strains (Figure 3A; Table S3). Strains CITA 14 and CITA 124, the pathogenic *Xap* strains CITA 33 and IVIA 2626.1 and the *Prunus*-non-virulent CITA 44, all isolated from *Prunus* spp. in Spain, shared 3,103 groups of homologous genes. A total of 889 cluster genes were found only in the non-virulent strains, while 708 cluster genes were found in the two Spanish strains of *Xap* (Figure 3B). Additionally, 236 CDS, 17 CDS, and 131 CDS, were unique for the pathovar *corylina, juglandis* and *pruni*, respectively (Table S3).

The mean of the gene content similarity among the analyzed genomes was 0.23 according to the Jaccard distance distribution, which means that the analyzed genomes shared a mean of 77% of their gene content, while the remaining 23% was unique for each one (Figure S3A).

Based in the gene cluster content of each genome, a principal component analysis showed that only 41.0% of the total difference was due to the variation by the two first principal components (Figure S3B). Three distinct clusters were elucidated, one of them was comprised by the *Prunus*-non-virulent or walnut strains (CITA 14, CITA 44, CITA 124, CFBP 7634 and CFPB 7651) and the strains with low-pathogenic-activity (3004, NCPPB 1630 and NCPPB 1832) which cause disease on barley and banana. The pathogenic strains of the pathovar *pruni* formed another cluster that comprised two subgroups, one formed by the strains isolated in Spain (CITA 33 and IVIA 2626.1) and another by the strains MAFF 301420 and MAFF 301427 isolated in Japan. Strains from the pathovar *juglandis* (CFBP 2528, CFBP 7179, Xaj2 and Xaj417) formed the third group. Finally, the strain NCCB 100457 from the pathovar *corylina* tended to group together with the strains isolated from walnut (Figure S3B).

**Figure 4:**
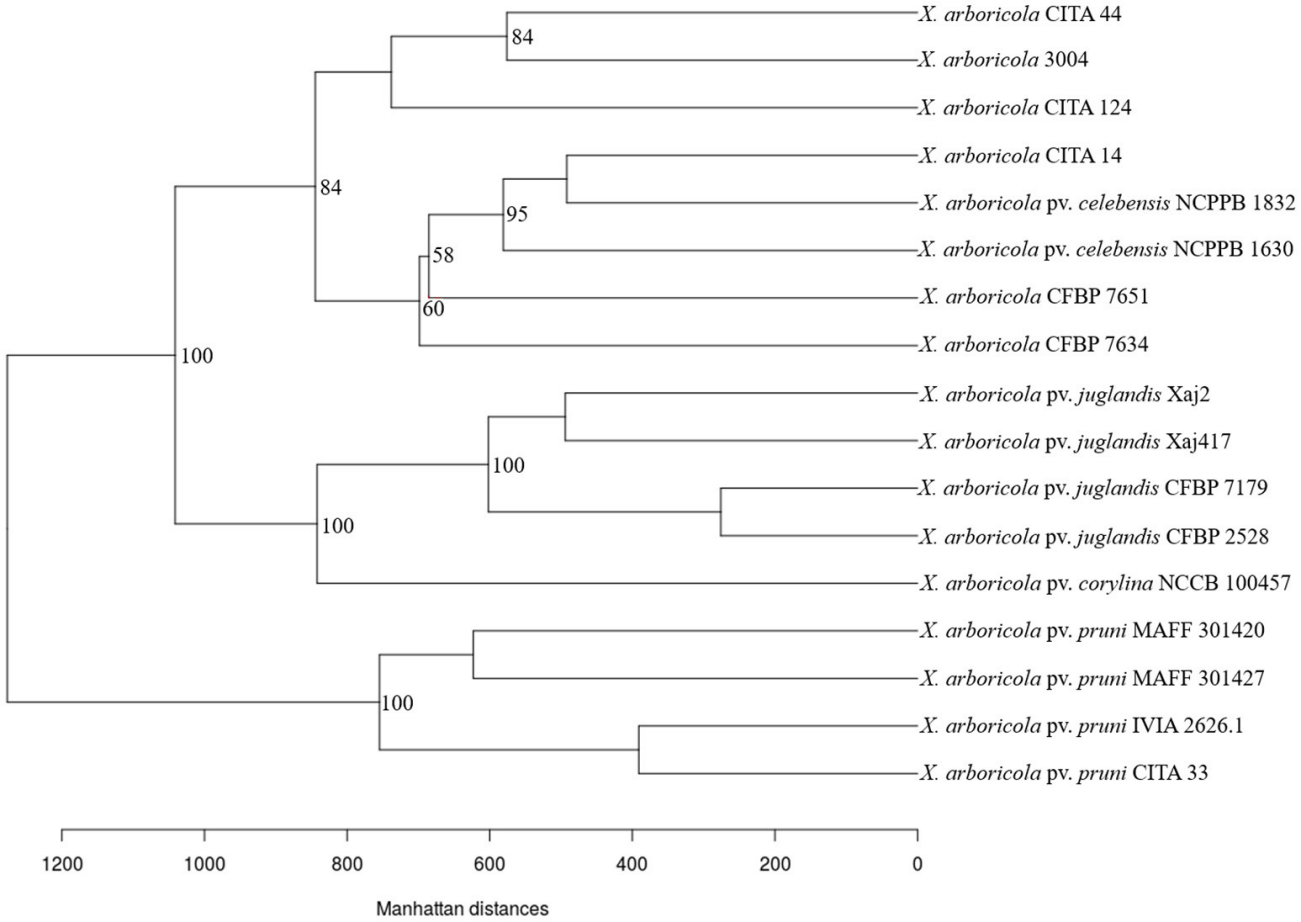
**Pan-genome tree for 17 strains of *Xanthomonas arboricola* with a variable virulence**. Tree construction was based in the distance between genomes according to the Manhattan distance. Bootstrap values over 50% are showed at the branch points.

The difference in the gene content cluster was illustrated using a pan-genome tree (Snipen and Liland, 2015) after computing the distance among the genomes using the Manhattan distance algorithm. The pan-genome tree for the 17 analyzed genomes (Figure 4) showed the same clustering organization that was visualized previously with the principal component analysis. Besides this, a division of the cluster comprised by the low-virulent and non-virulent strains was shown. A first group was composed by those strains that harbored components of the T3SS and T3Es, isolated from banana (NCPPB 1630 and NCPPB 1832), walnut (CFBP 7634 and CFBP 7651) and peach (CITA 14). A total of ten CDS differentiated this group from all the remaining clusters observed. The second group was comprised by the strain 3004 isolated from barley, and the strains CITA 44 and CITA 124 isolated from *Prunus*, and 27 CDS differentiated this cluster from all the other analyzed strains (Table S3). The same strains grouping and distribution was obtained using a Maximum likelihood phylogenetic analysis based in the concatenated sequence of the genes that comprised the core genome sequence of the 17 analyzed strains according to a sequence based methodology recently described (Page et al., 2015; Figure S4).

### Genes associated with pathogenicity in *X. arboricola* strains

In addition to the gene content comparison, the profiles of genetic components associated with pathogenesis were determined for CITA 14 and CITA 124 and compared to those in other strains of *X. arboricola* isolated from *Prunus* spp. in Spain, especially in the two pathogenic strains of *Xap*, CITA 33, and IVIA 2626.1, and in the *Prunus*-non-virulent strain of *X. arboricola* CITA 44.

Regarding the profiles of cell wall degrading enzymes, a total of ten genes that encoded for pectolytic enzymes were found in the five strains. CITA 14 and CITA 124 showed seven and eight of these genes, respectively. For these enzymes, two orthologs, NP_635517.1 and NP_635516.1, were shared by the *Prunus*-non-virulent strains, meanwhile the degenerated pectate lyase AAM37225.1 was only found in *Xap* strains CITA 33 and IVIA 2626.1 (Table S4). Regarding the profile of cellulolytic enzymes, nine of them were shared over 11 genes found in all the strains. In this case, only the presence of the cellulase AAM38359, described in *X. citri* subsp. *citri* 306, differentiated non-virulent strains from *Xap* (Table S4). In the case of the hemicellulolytic enzymes, a total of 11 orthologs were found in the *Prunus*-associated strains; pathogenic strains of *Xap* were differentiated from the non-virulent strains due to the presence of the genes that encoded the xylanase NP_638385.1 and the xylosidase/arabinosidase NP_637752.1, both described previously in *X. campestris* pv. *campestris* strain ATCC 33913. Finally, orthologous genes for the virulence associated lipases NP_638797.1 and AAO29541.1, from *X. campestris* pv. *campestris* ATCC 33913 and *Xylella fastidiosa* strain Temecula, were found in the five genomes (Table S4).

The profiles of genes related to sensing and chemotaxis varied among the analyzed strains. Four of the five Spanish *Prunus*-isolated strains presented the same gene profile for those genes associated with chemotaxis. However, CITA 124 did not harbor homologous genes to *cheD* (AAM36751.1), *cheZ* (AAM36793.1), and *cheA* (AAM36792.1), described in *X. citri* subsp. *citri* 306. Variants in other sensing mechanisms, such as TBDTs, were found in the *Prunus*-associated strains of *X. arboricola*. From the 17 TBDTs encoding genes found, those homologs of the proteins NP_635515.1, NP_635700.1, NP_635699.1 and NP_639391.1, initially described in *X. campestris* pv. *campestris* ATCC 33913, differentiated the atypical strains from *Xap*. Additionally, a large repertoire of 60 genes associated with STCRs was found in the analyzed genomes and, from them, 55 were shared for all the strains isolated from *Prunus*. In addition, the STCRs AAM36681.1, AAM35218.1, AAM37649.1 and NP_637535.1 were only present in the *Prunus*-non-virulent strains CITA 14, CITA 44, and CITA 124. Finally, from a total of 26 MCPs genes, 11 were found in all *Prunus*-associated strains, but the absence of an ortholog to CAJ23610.1, described in *X. campestris* pv. *vesicatoria* 85–10, in strains CITA 14 and CITA 124 differentiated them from the remaining strains (Table S4).

Besides to those genes related to environmental sensing, variations in some other genes associated with the initial steps of the pathogenesis process, such as motility, attachment, biopolymerization of the xanthan gum, and the inter-cellular cross-talk process controlled by the quorum-sensing system, were also found (Table S4). Pathogenic and non-virulent strains of stone fruit trees and almond shared 35 orthologs associated with molecular components of the flagellar system. The exception to this was the strain CITA 124, which did not have homologous genes to the flagellar components of *X. citri* subsp. *citri* 306, *flhF* (AAM36797.1), *fliH* (AAM36814.1), *fliJ* (AAM36812.1) and *motB* (AAM38537.1). In addition, an interesting polymorphism was observed in the flagellin protein, encoded by *fliC*, of non-pathogenic strains. In CITA 14 and CITA 124, this protein was identical to protein WP_024939608.1, which has been previously associated with all the non-virulent strains of *X. arboricola* or with low virulent strains of the pathovar *celebensis*. Pathogenic strains of *Xap* harbored a flagellin protein identical to protein WP_039814449.1, which present a substitution of aspartic acid for valine in the amino acid 43 of the N-terminal region that has been associated to pathogenic strains in other species of *Xanthomonas* (Sun et al., 2006; Cesbron et al., 2015).

Another bacterial structure related to motility, as well as to attachment, is the type IV pilus. The pathogenic *Xap* strains CITA 33 and IVIA 26262.1 harbored 25 orthologs to the 31 genes described in *X. citri* subsp. *citri*, while the atypical strains CITA 14, CITA 44, and CITA 124 were differentiated for the absence of orthologs to the genes *fimA* (AAM38084.1), *fimT* (AAM37516.1), *pilV* (AAM37515.1), *pilW* (AAM37514.1), *pilX* (AAM37513.1) and *pilY1* (AAM37512.1). With regard to the attachment function carried out by the non-fimbrial adhesins, most of the *Prunus*-non-virulent strains shared five of the six genes found, with the exception of CITA 14, which did not harbor a homolog to *fhaB1* of *X. campestris* pv. *vesicatoria* 85–10 (CAJ23537.1). Presence of a homologous gene to *fhaB2* (CAJ23538.1) of *X. campestris* pv. *vesicatoria* differentiated *Xap* from the atypical strains isolated from *Prunus* spp.

Pathogenic and non-virulent strains of *X. arboricola* isolated from *Prunus* spp. shared the same profile of xanthan-associated genes, which are involved in bacterial attachment and biofilm formation. None of the strains had homologous sequences to *gumG* (NP_637802.1) which was found in other xanthomonads (Lee et al., 2005). Regarding quorum sensing system, which is associated with the regulation of the pathogenic activity, all the analyzed strains, with the exception of CITA 14, harbored the same gene pattern conformed by 11 of the 12 genes associated to this process in *Xanthomonas* (He and Zhang, 2008). In addition, CITA 14 harbored an ortholog to the transcriptional regulator NP_636589.1 described in *X. campestris* pv. *campestris* ATCC 33913 (Table S4).

Bacterial type II, III, and IV secretory systems (T2SS, T3SS, and T4SS), which are related to the secretion of proteins and DNA, also play a crucial role in pathogenesis (Ryan et al., 2011). Regarding to T2SS, CITA 14 and CITA 124 presented 19 and 18 orthologs, respectively, of the 23 genes associated with *xcs* and *xps* T2SS gene clusters described in *Xanthomonas* (Filloux, 2004; Szczesny et al., 2010). The only difference among atypical strains and *Xap* was the presence in the latter of an ortholog to the gen *xcsK* (NP_638764.1; Table S4).

Pronounced differences were observed among pathogenic and non-virulent to *Prunus* strains regarding the gene profile associated with T3SS and its related effectors, as well as with T4SS. T3SS-related gene profile in CITA 14 was comprised by 24 orthologs of the 28 T3SS described in *Xanthomonas*, and this profile was the same observed in pathogenic strains of *X. arboricola* (Cesbron et al., 2015; Garita-Cambronero et al., 2016a). On the other hand, CITA 124 only harbored four T3SS-related genes (*hpaS, hpaR2, hrpG* and *hrpX*) which correspond to the regulators of this secretory system (Jacobs et al., 2015). For this strain, as for the *Prunus*-non-virulent strain CITA 44, none of the genes that composed the macromolecular structure of the T3SS were found (Table S3). Regarding to the T3SS related effectors, from the 61 T3Es and other T3SPs described in *Xanthomonas*, the genome sequence of CITA 14 presented a total of six orthologs to the genes *avrBs2, hpaA, hrpW, xopA, xopF1* and *xopR*, while genome sequence of CITA 124 did not present any of these effectors. Moreover, *X. arboricola* strains isolated from *Prunus* showed variants in the number of T4SSs. Most of the strains, regardless of their pathogenic activity, contained ten of the 12 components associated with the VirB/VirD4 T4SS of *Agrobacterium tumefaciens* (Christie, 2004). In addition, the absence of orthologs to the core components associated with the type IV conjugation cluster *tfc*, described in *Haemophilus influenzae*, in the strains CITA 14 and CITA 124, differentiated them from the *Xap* strains.

Finally, comparative sequence analysis among the nucleotide sequence of the plasmid pXap41 and the draft genome sequence corroborated the absence of this plasmid in both non-virulent strains (Figure S5).

### The real-time PCR test for *xopE3* permitted to differentiate *Xap* from atypical strains of *X. arboricola* isolated from *Prunus* spp.

*In silico* analysis of the primers XopE3F (5′-TCAGCGATCACGCATCCA-3′), XopE3R (5′-CGCACCAGATCGACAAACAC-3′) and the probe XopE3p (5′-6-carboxyfluorescein [FAM]-CATGCGCAGGCCGCACAT-[TAMRA]-3′), indicated that they were able to amplify the gene *xopE3* in *X. arboricola* only in those sequences from the pathovar *pruni*. Sequence analysis on the available complete genome sequences of *X. arboricola* showed that *xopE3* was only present in those strains of the pathovar *pruni* (Figure S6). In addition, this set of primers and the designed probe were also able to amplify the *xopE3* gene in other species of *Xanthomonas* such as one strain of *X. campestris* (IVIA 2734.1), one strain of *X. citri* subsp. *citri* (306) and *X. fuscans* subsp. *fuscans* strains NCPPB 381 and IVIA 151835DA (Table S1).

Besides the nucleotide sequence-based analysis, the specificity of the real-time PCR assay was conducted by testing the protocol on the bacterial strains listed in Table S1. Among the *X. arboricola* strains, only those identified previously as *Xap*, by the presence of the plasmid pXap41, and with a positive result for the standardized real-time protocol based in the gene *ftsX* of the ABC transporter in *Xap* (Palacio-Bielsa et al., 2011, 2015), presented consistent positive results. None of the seven *Xap*-look-a-like strains (CITA 14, CITA 42, CITA 44, CITA 49, CITA 51, CITA 124 and CITA 149) showed positive results from this PCR. Undesired specific PCR results for *xopE3* were observed from one strain of *X. campestris* (IVIA 2734-1), three strains of *X. citri* subsp. *citri* (306, IVIA 2889-1 and IVIA 3026-1), one strain of *X. hortorum* pv. *pelargonii* (CITA Xp-2), two strains of *X. fuscans* subsp. *fuscans* (NCPPB 381 and IVIA 151835DA), and the strain IVIA 3619-1 of *X. vesicatoria* (Table S1). When the analyzed strains were amplified using the real-time PCR protocol for the ABC transporter-associated gene *ftsX* (Palacio-Bielsa et al., 2011, 2015), positive results were obtained with all the strains of *Xap*, the seven strains of *Xap*-look-a-like, two strains of *X. arboricola* pv. *corylina* (CFBP 1846 and IVIA 3978) and the strain of *X. citri* subsp. *citri* 306. Double positive PCR results, using *xopE3* or ABC primers, were only observed for all the *Xap* strains but also for the strain 306 of *X. citri* subsp. *citri* which is unlikely to be found in *Prunus* spp. (Table S1).

**Figure 5:**
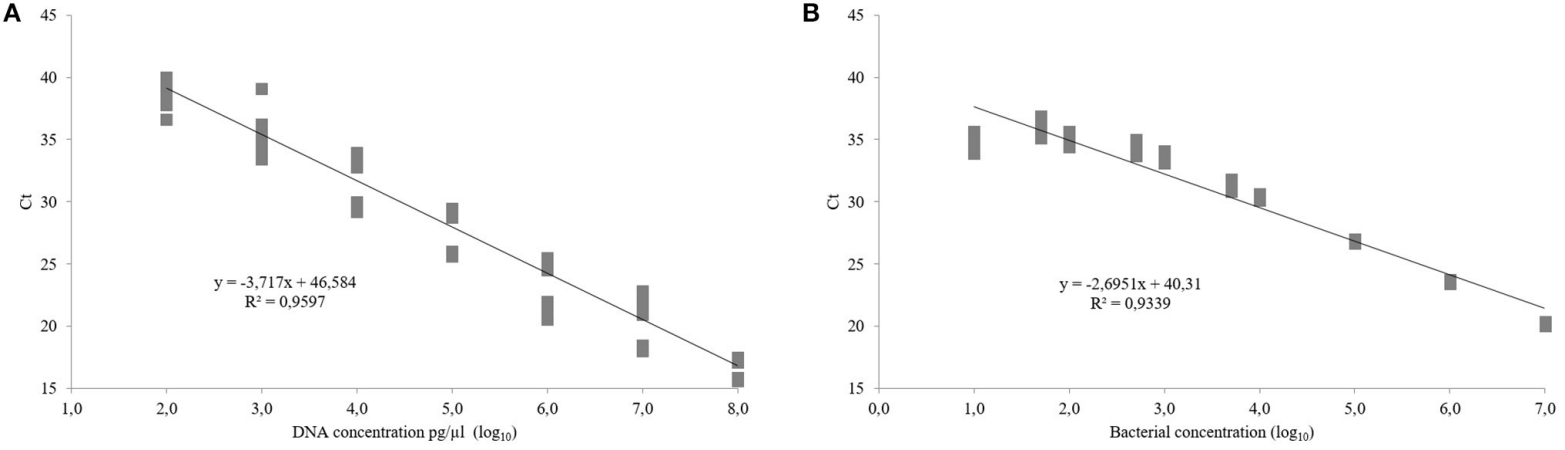
**Calibration curves for detection of *xopE3* in *Xanthomonas arboricola* pv. *pruni***. Calibration curves have been obtained from dilution series of purified DNA **(A)** and bacterial cells **(B)** of *X. arboricola* pv. *pruni* strain CITA 33. Real-time PCR amplification was performed in three independent assays using the primers XopE3F/R and the TaqMan probe XopE3p.

No significant differences were found among the three independent assays conducted to determine the sensitivity of the real-time PCR protocol to amplify *xopE3* using heat-treated cells or purified DNA as samples. Calibration curves, obtained from serial dilutions of heat-treated cells of *Xap* strain CITA 33, demonstrated that the real-time PCR assay showed a sensitivity of 10 CFU/ml or 100 pg/μl of DNA, with a PCR efficiency of 2.2 ± 0.22 or 1.8 ± 0.03 for bacterial cells or purified DNA, respectively (Figure 5).

## Discussion

The results obtained in the initial characterization of the *X. arboricola* strains isolated from *Prunus* spp. pointed out that one of the most widely used real-time PCR protocol for detecting *Xap* (Palacio-Bielsa et al., 2011, 2015) was not able to differentiate bacterial strains of this pathovar from those atypical strains of the same species, which are part of the *Prunus* microbiota. Actually, *in silico* analysis, based on the nucleotide sequence comparison among the available genome sequences of *X. arboricola* and the target genomic regions proposed for the identification of *Xap* in a variety of other published PCR protocols (Park et al., 2010; Pothier et al., 2011a; Figure S7), demonstrated that none of them could be able to discriminate between these two groups of *Prunus*-associated strains.

The MLSA analysis, conducted with the housekeeping genes *dnak, fyuA, gyrB* and *rpoD*, resulted in useful to characterize typical and atypical strains of *X. arboricola* as proposed in recent articles (Essakhi et al., 2015; Garita-Cambronero et al., 2016a) and corroborated the existence of genomic variants among the atypical strains of *X. arboricola* isolated from *Prunus*. As described previously for the genes used in this MLSA scheme, the phylogenetic clustering of the MLSA did not correspond to the phylogenetic arrangement based on individual locus. In this case, it was observed that the atypical strains of *X. arboricola* isolated from *Prunus* spp. were scattered on the phylogenetic tree. This disagreement has been associated with the probable existence of recombination events that shuffle the phylogenetic signal and also by the fact that each locus, individually, does not harbor enough phylogenetic information (Essakhi et al., 2015; Fischer-Le Saux et al., 2015).

An initial evaluation of the pathogenic activity of the seven atypical strains of *X. arboricola* detected by MLSA revealed variations in their virulence; for instance, all of them cause necrosis on the susceptible peach rootstock GF-305, but after 21 dpi their populations in the inoculated leaves decreased reflecting a non-compatible plant-microbe interaction described also for other *Xanthomonas* (Ah-You et al., 2007). Therefore, these strains must be considered non-virulent in this host. The interaction of these atypical strains with other host plants showed differences among strains and hosts; for example, all atypical strains, with exception of CITA 44, showed a necrotic spot in the infiltrated tomato leaf-zone, while only CITA 14 and CITA 149 were able to cause necrosis on *Nicotiana* spp. These variations concurred with the results obtained previously in a linage of non-virulent strains of *X. arboricola* isolated from *Juglans regia* (Essakhi et al., 2015). As in that study, the linage of non-virulent *X. arboricola* strains isolated from *Prunus* spp. (Hajri et al., 2012; Cesbron et al., 2015; Garita-Cambronero et al., 2016a) showed a non-canonical T3SS and T3Es repertoire in CITA 14 and CITA 149, or the absence of T3SS and T3Es, as in strains CITA 42, CITA 49, CITA 51 and CITA 124.

A global overview of these results led us to the question why, even in the absence of the canonical T3SS or the T3Es described in such strains (White et al., 2009), some of them were able to cause hypersensitive response on *Nicotiana* spp., tomato and the peach rootstock GF-305, while others like strain CITA 44 did not cause apparent effect on the assayed hosts.

Due to the fact that these variants could not be clarified only based on the PCR typing of the components for the T3SS and its related effectors, a more in-depth analysis based on other pathogenicity determinants that could play a role in this plant-microbe interaction was needed. Consequently, a whole-genome comparative analysis was performed on the strains CITA 14, CITA 44, and CITA 124, which were representatives of the three MLSA clusters that enclosed non-virulent strains isolated from *Prunus*.

Whole genome sequencing of these three strains permitted us to accurately infer their phylogenetic position within *X. arboricola*. After a comparative analysis of the groups of orthologous genes found in the pan-genome of *X. arboricola*, it was possible to infer a clear pathovar-based clustering of the strains, as reported previously in other strains of *X. arboricola* isolated from *Juglans regia* with non-canonical T3SS. The *Prunus*-non-virulent strains CITA 14 and CITA 124, isolated from *P. persica*, were closely related to those strains of *X. arboricola* that do not cause disease (CFBP 7634, CFPB 7651 and CITA 44; Cesbron et al., 2015; Garita-Cambronero et al., 2016a), or have a low virulent ability (3004, NCPPB 1630 and NCPPB 1832; Ignatov et al., 2015; Harrison et al., 2016). Additionally, a phylogenetic analysis based in the concatenated nucleotide sequences of all the genes shared by the studied strains has corroborated the assignment of CITA 14 and CITA 124 to a cluster that included the strains mentioned above, which is located in a basal phylogenetic position within the species *X. arboricola*.

The knowledge about the variable distribution of the T3SS and its related secreted proteins between the pathogenic and the low or non-virulent groups of *X. arboricola* could provide insights regarding the acquisition of pathogenicity in *Xanthomonas* (Jacobs et al., 2015). It could be possible that in *X. arboricola*, after the acquisition of the master regulators of the T3SS and related proteins, the initial acquisition of some T3Es could led to the emergence of generalist pathogenic strains, revealed here in the phylogenetic group composed by low-pathogenic strains on a wide host range, whereas the subsequent acquisition of novel T3Es could shape the specialization of the most pathogenic pathovars on their specific host range (Jacques et al., 2016). Despite the fact that in this study we did not have conclusive results in this matter, it could be interesting to perform future comparative and evolutionary studies to test this general hypothesis of the genus, for which *X. arboricola* could be a good subject of study.

Genome comparative analysis also showed variants among CITA 14, CITA 124 and the available genomes of *X. arboricola* in a large list of genes that have been associated with different stages of the pathogenic process in *Xanthomonas* spp. (Table S4). On one hand, in these two strains, slight differences, related to environmental sensing such as the MCPs, TBDTs and the STCRs, were found. On the other hand, major differences were found in those features associated with the flagellin protein sequences, as well as with the molecular components of the type IV pilus. In other xanthomonads, the flagellin polymorphism, observed here between virulent and atypical strains from *Prunus*, has been associated with the ability of the plant to detect the bacteria and to trigger the plant immune response associated with the initial stages of the plant-pathogen interaction (Sun et al., 2006).

In *Xanthomonas*, the type IV pilus seems to play an important role in bacterial host-interaction and pathogenesis, in twitching motility, in the formation of mature biofilms and in the interaction with bacteriophages (Dunger et al., 2016). In *X. arboricola* all the described non-virulent strains and strains CITA 14 and CITA 124 (Cesbron et al., 2015; Garita-Cambronero et al., 2016a) showed a gene arrangement similar to the one previously observed in *X. translucens* pv. *undulosa* strain Xtu 4699, which is characterized by the absence of homologs of *fimA, fimT, pilV, pilW, pilX* and *pilY1* (Dunger et al., 2016). In the *Prunus*-non-virulent strain CITA 44, the absence of these minor pilins does not alter the twitching type motility (Garita-Cambronero et al., 2016a). From all the variants found in the molecular components of this macromolecular structure, only mutants in the orthologue of *pilY1* have shown a reduction in virulence in the non-vascular pathogen *X. oryzae* pv. *oryzicola* (Burdman et al., 2011). In all the pathogenic pathovars of *X. arboricola*, included the strain NCPPB 1630 of the pathovar *celebensis*, homologs of the minor pilins mentioned above were found, but all of them showed a percentage of identity lower than 80% with respect their orthologues in *X. citri* subsp. *citri* 306 (Dunger et al., 2014).

As reported in previous studies (Cesbron et al., 2015; Essakhi et al., 2015; Garita-Cambronero et al., 2016a), remarkable differences have been found among pathogenic and low or non-virulent strains of *X. arboricola* with respect to the T3SS and T3Es. Non-virulent and low-virulent strains of this species were separated in two different groups, one of them composed by those strains described in *X. arboricola* (CITA 14, CFBP 7651, NCPPB 1630 and NCPPB 1832), isolated from banana, stone fruit trees or walnut, that harbored the molecular components of the T3SS and shared the six core T3Es, *avrBs2, hpaA, hrpW, xopA, xopF1* and *xopR*. One exception to this group was strain CFBP 7634, isolated from walnut, which only harbored two of the T3Es, *xopR* and *avrBs2*, and did not possess homologs for the T3SS (Cesbron et al., 2015). It would be interesting to determine if the strains isolated from *Junglans* and *Prunus* are able to cause disease on banana performing pathogenicity tests in tropical conditions. A second group, comprised by the *Prunus*-non-virulent strains CITA 44 and CITA 124, isolated from *Prunus* spp., and the pathogenic strain 3004, isolated from barley, was characterized by the absence of T3SS and T3Es. Due to the fact that these strains are closely related according to the phylogenetic analysis, pathogenicity of CITA 44 and CITA 124 was tested on barley, but negative results obtained pointed out that the ability of strain 3004 to cause disease on this host could be related to other features that are not shared among this strain and strains CITA 44 and CITA 124.

In addition to the flagellin polymorphism mentioned above and its possible role on the plant immune response, a recent study on *X. euvesicatoria* described 17 T3Es that inhibit the plant immunity triggered (PTI) by the domain flg22 in *Arabidopsis thaliana* (Popov et al., 2016) and, from these, the T3Es *xopB, xopE2, xopF1, xopL, xopN, xopV, xopX* and *xopZ* have been predicted in *X. arboricola*. Those pathogenic strains that cause disease on hazelnut, stone fruit trees and walnut presented seven of these T3Es, while the non-pathogenic CFBP 7651, the *Prunus*-non-virulent CITA 14, and the banana-pathogenic strains NCPPB 1630 and NCPPB 1832, only harbored the T3E *xopF1*. Further functional studies comprising these PTI inhibitors would be useful to understand their role to sidestep the initial plant defense mechanisms and the development of a compatible plant-pathogen interaction. For these purposes, the use of non-virulent strains, such as CITA 14, could be useful to determine if these T3Es are playing a key role inhibiting the PTI in *Prunus* spp.

Regarding the type IV secretion systems, which are molecular structures adapted to translocate large molecules like proteins or protein-DNA complexes through multiple cell membranes (Guglielmini et al., 2014), the VirB/VirD4 system has been found in all the strains of *X. arboricola*. Nevertheless, the profile of proteins associated with this T4SS was almost the same in all the strains, with the exception of the homologs of VirB6, which is scattered within the members of the *X. arboricola*. According to the studies of mutants of *virB6* in *A. tumefaciens*, this gene is essential for the biogenesis of the T pilus and the secretion channel (Jakubowski et al., 2003); but in *X. citri* subsp. *citri* and in *X. campestris* pv. *campestris*, this T4SS has been described as not playing a main role in virulence (Jacob et al., 2014). Given that the presence of the complete core of components for its expression in *X. arboricola* varies among strains, regardless of the pathogenic activity, the VirB/VirD4 secretion system may not be essential for virulence in this species. Despite this, functional experiments with T4SS-deleted mutants are required for testing this hypothesis.

Evidence of a group of genes putatively related to the *tfc* T4SS of *H. influenzae*, which is related to bacterial conjugation, have been obtained after searching for homologs using the Blast tool from the NCBI, and also corroborated using the web-based tool for prediction of T4SS-related genes T346Hunter (Martínez-García et al., 2015); but the identity of the putative orthologous genes found in *X. arboricola* showed an amino acid sequence identity lower than 80% for all the genes. Despite of this, the presence of this group of genes, annotated as integrating conjugative elements, varied among *X. arboricola* strains and were only present in those pathogenic organisms from the pathovars *juglandis* and *pruni*.

As a final result of this comparative analysis, the absence of the recalcitrant plasmid pXap41 observed by the multiplex-PCR approach (Pothier et al., 2011b) was corroborated in CITA 14 and CITA124 and, as proposed previously, it was only found in *X. arboricola* pv. *pruni*. Presence of this plasmid has been useful not only to differentiate such pathovar from the other pathovars of *X. arboricola* as proposed previously (Pothier et al., 2011b), but also to distinguish pathogenic strains of *Xap* from other strains of *X. arboricola* that cohabit *Prunus* spp. In addition to this feature, the pan-genomic analysis pointed out a series of unique genes for each infrasubspecific group of *X. arboricola* that could be interesting targets for developing new precise diagnostic tools.

Due to the fact that this plasmid contains at least three virulence factors, *xopAQ, xopE3* and *mltB*, which in *X. arboricola* are unique in pathovar *pruni*, it was confirmed to be a good target for conducting studies of host specialization in the *Xap-Prunus* relationship. Additionally, it is useful as a genomic marker to differentiate *Xap* from all the other members of the species, especially from those atypical strains found in *Prunus* spp.

In this work, the use of pXap41, specifically a partial sequence of the virulence-associated gene *xopE3*, was explored for designing a sensitive and specific real-time PCR-based test to differentiate *Xap* from other *X. arboricola* strains. As shown here, the developed test was highly sensitive on both heat-treated bacterial cells and purified DNA, but showed unwanted positive amplification in one strain of *X. campestris*, three strains of *X. citri* susbp. *citri*, two strains of *X. fuscans* susbsp. *fuscans*, one strain of *X. hortorum* pv. *pelargonii*, and one strain of *X. vesicatoria*. To our knowledge, there is not record of the presence of these species on *Prunus* spp., and consequently to find one of them in natural conditions on these hosts is unlikely or possible only as a fortuitous event. The previously developed real-time PCR for detecting *Xap* (Palacio-Bielsa et al., 2011, 2015), as well as the other PCR-based methods designed for this purposes, with the exception of the Bio-PCR protocol proposed by Ballard et al. (2011), were not able to differentiate those members of the pathovars *corylina* and *pruni* (Figure S7). But the most important problem was that as has been shown here, the methods described were not able to differentiate *Xap* from non-virulent strains found in *Prunus* spp. Therefore, based in our results we suggest a real-time PCR amplification protocol based in *xopE3* gene for *Prunus*-isolated strains that could be used in conjunction with the method proposed by Palacio-Bielsa et al. (2011, 2015) for routine detection and identification of this quarantine pathogen, causal agent of the bacterial spot of stone fruit trees and almond. A combined result of both tests gives a precise identification of the xanthomonads detected in *Prunus* spp. If both tests result positive, the bacterial isolate could be identified as *Xap* and, on the other hand, if the isolated bacterium shows positive results only for the ABC-method it could be designated as member of the *Xap*-look-a-like group. Both, the multiplex conventional PCR described by Pothier et al. (2011b), and the combination of two real-time PCR protocols, proposed here, are suitable to differentiate *Xap* strains. However, the latter offers advantages because it allows detecting *Xap* from plant material (including asymptomatic samples; Peñalver et al., 2016), whereas the protocol proposed by Pothier et al. (2011b) has only been assayed using pure bacterial cultures. *Xanthomonas* group associated to *Prunus* spp. requires further taxonomic analyses for more accurate description of the taxonomic status of the different strains. Exploration of the transcriptome and the metabolome of such strains could also help in identifying factors contributing to their diversity.

There are a small number of pan-genomes for species of plant pathogenic bacteria now available. Moreover, studies performed in other bacterial species have shown that it is compulsory to analyse multiple genomes to get an overall picture of the bacterial group studied. As a whole, the pan-genome of *X. arboricola* and the characterization of the atypical *X. arboricola* strains found on *Prunus* spp., as well as their use to study the genomic diversity of *X. arboricola*, has revealed and corroborated the existence of a distinct phylogenetic basal lineage of this species which is associated with a wide host range. From the strains included in this group, those considered as low-virulent seemed to cause disease in two species of monocotyledon plants (banana and barley). After an extensive comparative analysis of those virulence-related genes, it was determined that this bacterial lineage slightly differed from those which are considered as highly virulent in several features associated with the initial or later stages of the pathogenicity process.

The genomic analysis performed in this work not only reveals a series of genes potentially implicated in the pathogenesis of *X. arboricola* pv. *pruni* on *Prunus* spp. but also has a practical implication in the disease control of the bacterial spot of stone fruit trees and almond, providing a new tool for its diagnosis. Finally, to improve the knowledge on the pathogenic ability and diversity of the bacteria from this species will eventually open the way for the development of innovative control strategies for the diseases caused by them.

## Author contributions

Wrote the paper, Conceived and designed the experiments: JG, AP, ML, and JC. Performed the experiments: JG, AP, and JC. Analyzed the data: JG and JC.

## Funding

This work was supported financially by the Instituto Nacional de Investigación y Tecnología Agraria y Alimentaria (INIA) project RTA2014-00018. JG held a Ph.D. fellowship from the Spanish Government (Ministerio de Educación, Cultura y Deporte; fellowship FPU12/01000).

### Conflict of interest statement

The authors declare that the research was conducted in the absence of any commercial or financial relationships that could be construed as a potential conflict of interest.

## Supplementary material

Figure S1 **Maximum likelihood trees based on partial sequences of *dnaK, fyuA, gyrB*, and *rpoD***. Bootstrap values (1,000 replicates) are indicated over or below the branches.

Figure S2 **Schematic representation of bacterial-caused symptoms on *Prunus persica* (GF-305), *Hordeum vulgare, Nicotiana benthamiana, Nicotiana tabacum*, and *Solanum lycopersicum* 21 dpi**. Leaves were infiltrated with 10^8^ CFU/mL of bacteria or with sterile phosphate saline buffer (PBS) as negative control.

Figure S3 **Comparative statistics among strains of *Xanthomonas arboricola* based in the distribution of the potential orthologous clusters of gene contained in the genome sequence of 17 bacterial strains**. Histogram representing the Jaccard distance distribution among the analyzed genomes **(A)**. Principal component analysis based in the potential orthoglogous clusters gene present in the pan-genome of *X. arboricola* showing how the genomes are located in the space spanned by the two first principal components **(B)**.

Figure S4 **Phylogenetic analysis of 17 strains of *Xanthomonas arboricola* based on the core genome sequence (2,714 potential groups of orthologous genes) and representation of the distribution of the potential orthologous cluster genes of the pangenome (7,074) within the analyzed genome sequences**. Sequences were aligned using PRANK and maximum likelihood analysis was carried out using RaxML. Bootstrap values (1,000 replicates) are presented above or below the branches.

Figure S5 **Presence of the plasmid pXap41 in the genome-sequenced strains of *Xanthomonas arboricola***. Comparative sequence analysis was performed using Blastn with an expected value threshold of 0.001 and graphically represented by the BRIG tool. Each concentric circle represents one of the analyzed genomes.

Figure S6 ***In silico* representation of the presence of *xopE3* and *ftsX* and the hybridization zone for the primers and probes used for real-time PCRs amplification in *Xanthomonas arboricola***. Comparative sequence analysis was performed using Blastn with an expected value threshold of 0.001 and graphically represented by the BRIG tool. Each concentric circle represents one of the analyzed genomes.

Figure S7 ***In silico* representation of the hybridization zone for the primers used in two PCR amplification protocols published to identify *Xanthomonas arboricola* pv. *pruni***. Comparative sequence analysis was performed using Blastn with an expected threshold value of 0.001. The circular graphic has been constructed using BRIG. Each concentric circle represents one of the analyzed genomes.

Table S1 **Bacterial strains used in this study**.

Table S2 **Genome statistics of 17 strains of *Xanthomonas arboricola* with a variable virulence**.

Table S3 **Unique clusters of orthologous genes encoded in the genome sequence of the non-virulent to *Prunus* strains of *Xanthomonas arboricola* CITA 14 and CITA 124 as well as those encoded in the pathogenic strains of the pathovars *corylina, juglandis* and *pruni***.

Table S4 **Orthologous protein coding sequences (CDS) of 17 strains of *Xanthomonas arboricola* associated with pathogenesis**.
