# Supplementary figures and images for: Pan-Genomic Analysis Permits Differentiation of Virulent and Non-virulent Strains of Xanthomonas arboricola That Cohabit Prunus spp. and Elucidate Bacterial Virulence Factors
Source: Front Microbiol. 2017 Apr 13;8:573. doi: 10.3389/fmicb.2017.00573 (PMC5389983; doi:10.3389/fmicb.2017.00573)

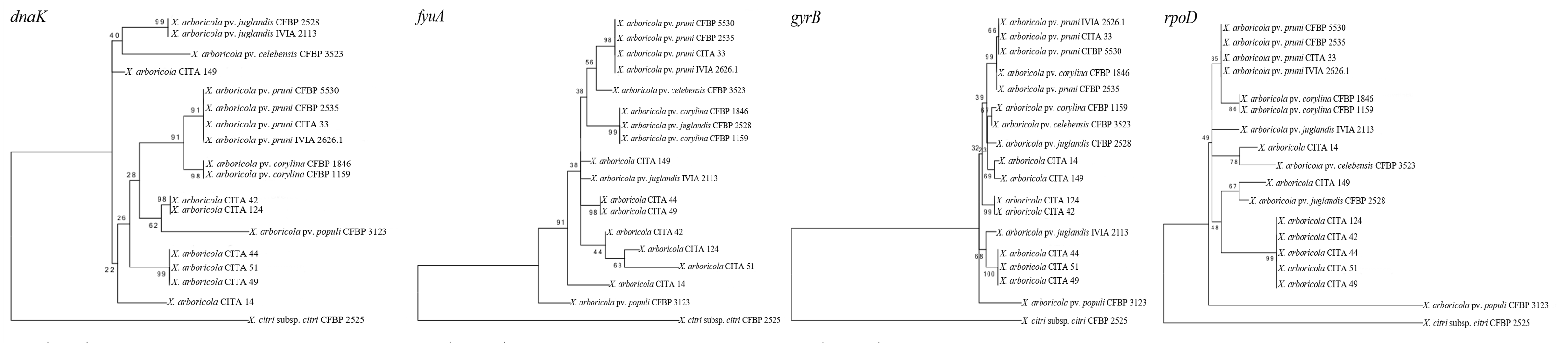

Supplement: Figure S1 — Maximum likelihood trees based on partial sequences of dnaK, fyuA, gyrB, and rpoD. Bootstrap values (1,000 replicates) are indicated over or below the branches. [file Image1.TIF]

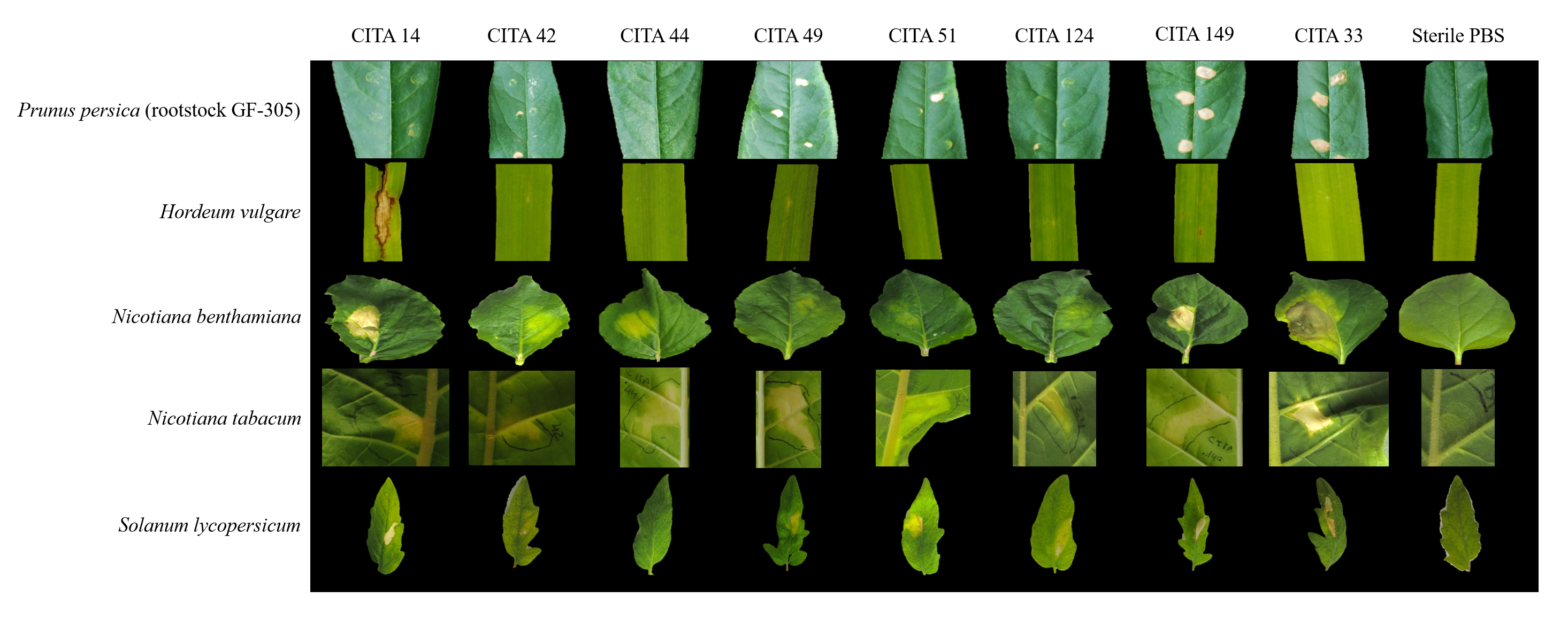

Supplement: Figure S2 — Schematic representation of bacterial-caused symptoms on Prunus persica (GF-305), Hordeum vulgare, Nicotiana benthamiana, Nicotiana tabacum, and Solanum lycopersicum 21 dpi. Leaves were infiltrated with 108 CFU/mL of bacteria or with sterile phosphate saline buffer (PBS) as negative control. [file Image2.TIFF]

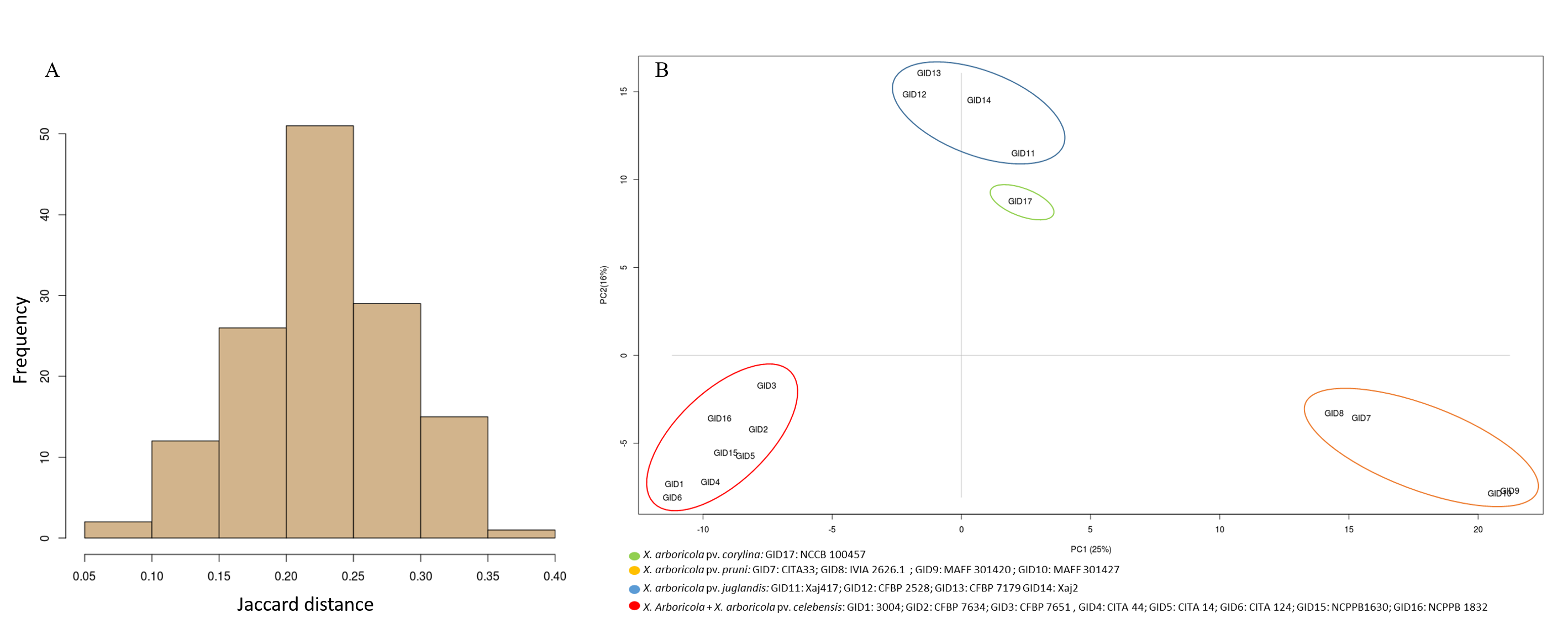

Supplement: Figure S3 — Comparative statistics among strains of Xanthomonas arboricola based in the distribution of the potential orthologous clusters of gene contained in the genome sequence of 17 bacterial strains. Histogram representing the Jaccard distance distribution among the analyzed genomes (A). Principal component analysis based in the potential orthoglogous clusters gene present in the pan-genome of X. arboricola showing how the genomes are located in the space spanned by the two first principal components (B). [file Image3.TIFF]

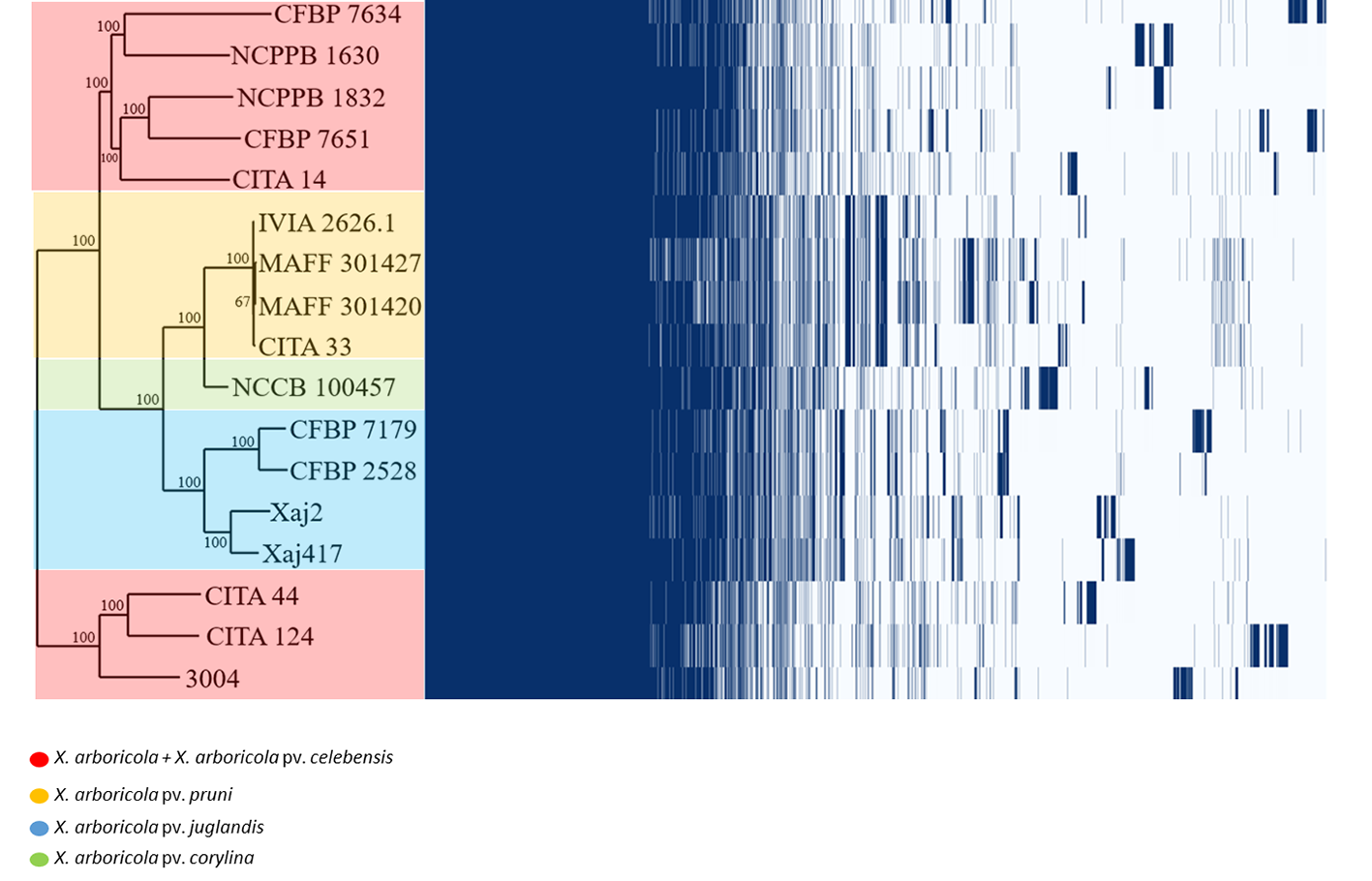

Supplement: Figure S4 — Phylogenetic analysis of 17 strains of Xanthomonas arboricola based on the core genome sequence (2,714 potential groups of orthologous genes) and representation of the distribution of the potential orthologous cluster genes of the pangenome (7,074) within the analyzed genome sequences. Sequences were aligned using PRANK and maximum likelihood analysis was carried out using RaxML. Bootstrap values (1,000 replicates) are presented above or below the branches. [file Image4.TIFF]

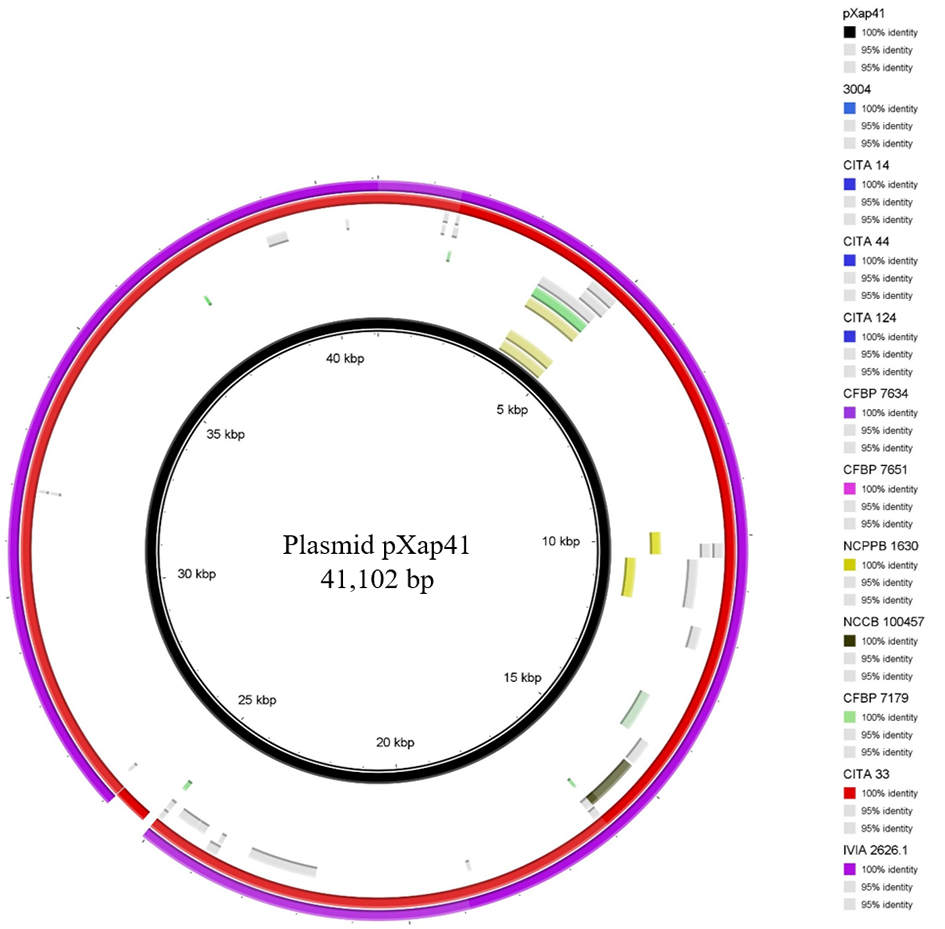

Supplement: Figure S5 — Presence of the plasmid pXap41 in the genome-sequenced strains of Xanthomonas arboricola. Comparative sequence analysis was performed using Blastn with an expected value threshold of 0.001 and graphically represented by the BRIG tool. Each concentric circle represents one of the analyzed genomes. [file Image5.TIFF]

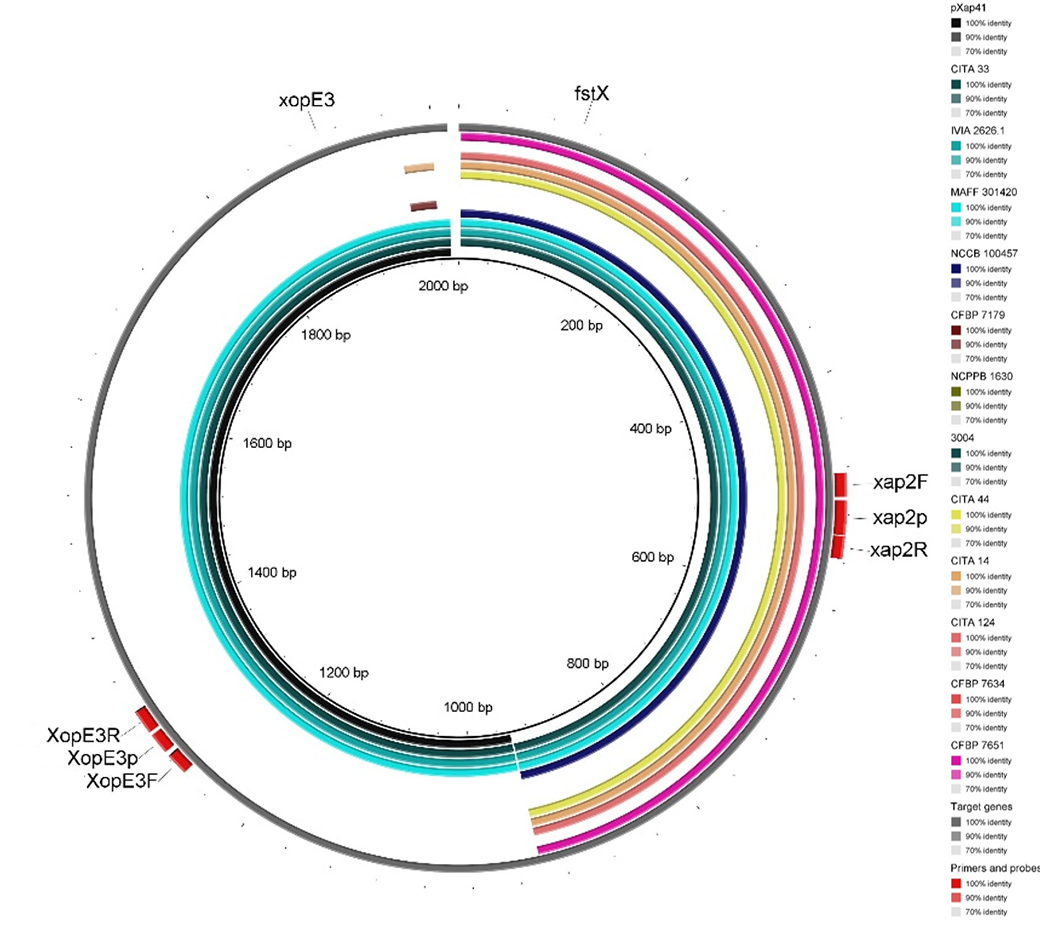

Supplement: Figure S6 — In silico representation of the presence of xopE3 and ftsX and the hybridization zone for the primers and probes used for real-time PCRs amplification in Xanthomonas arboricola. Comparative sequence analysis was performed using Blastn with an expected value threshold of 0.001 and graphically represented by the BRIG tool. Each concentric circle represents one of the analyzed genomes. [file Image6.TIFF]

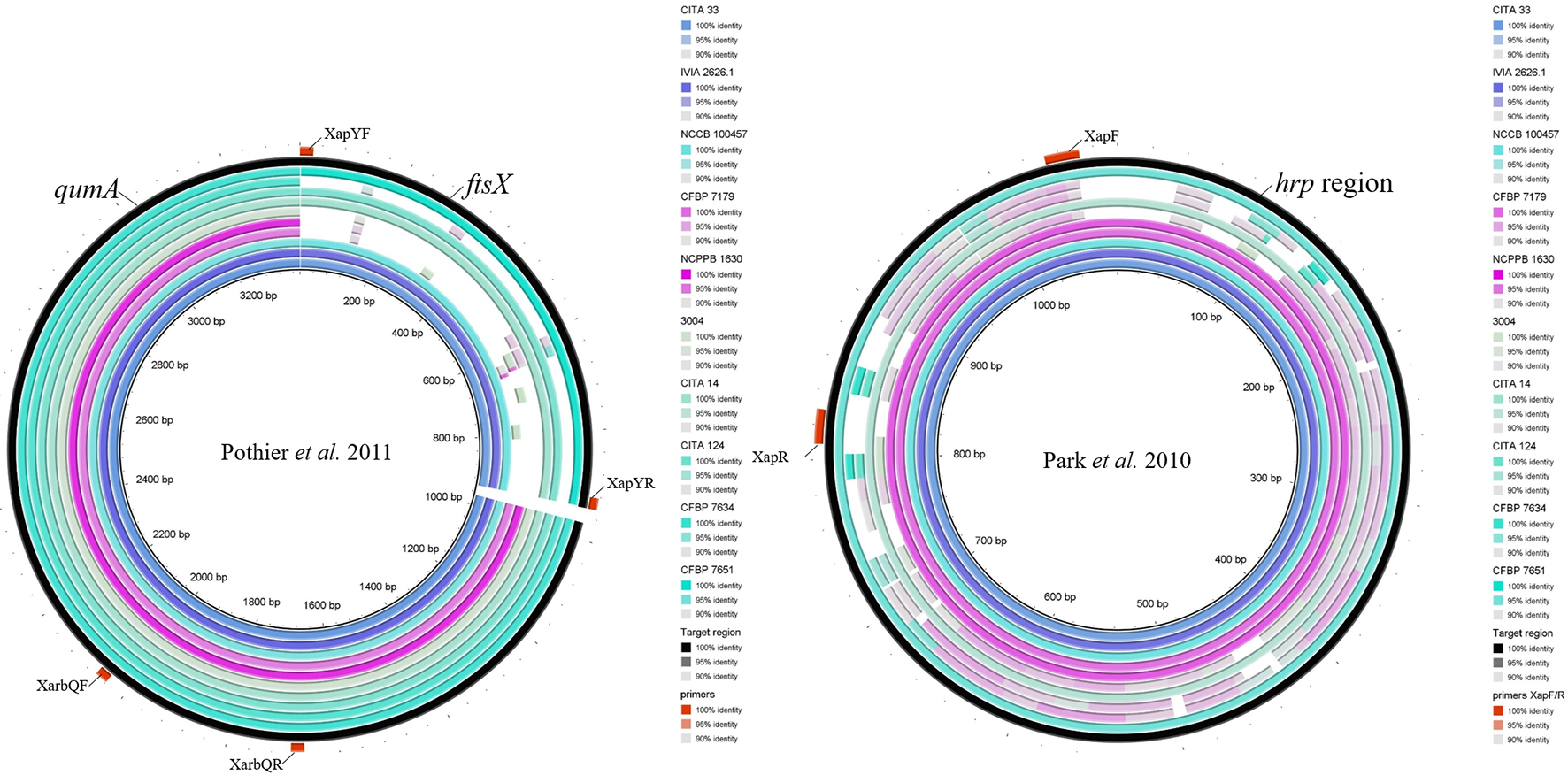

Supplement: Figure S7 — In silico representation of the hybridization zone for the primers used in two PCR amplification protocols published to identify Xanthomonas arboricola pv. pruni. Comparative sequence analysis was performed using Blastn with an expected threshold value of 0.001. The circular graphic has been constructed using BRIG. Each concentric circle represents one of the analyzed genomes. [file Image7.TIFF]
