# Supplementary material for: Pan-Genomic Analysis Permits Differentiation of Virulent and Non-virulent Strains of Xanthomonas arboricola That Cohabit Prunus spp. and Elucidate Bacterial Virulence Factors
Source: Front Microbiol. 2017 Apr 13;8:573. doi: 10.3389/fmicb.2017.00573 (PMC5389983; doi:10.3389/fmicb.2017.00573)
Supplement: Table S1 — Bacterial strains used in this study. [file Table1.pdf]

Table S1. Bacterial strains used in this study

Taxa	Strain	Origin	Host	*ftsx*	*xopE3*	pXap41
Xanthomonas arboricola						
X. arboricola pv. *pruni**	100.343	Zuidwolde (Netherlands)[b]	*Prunus laurocerasus* cv. Caucasica	+	+	+
X. arboricola pv. *pruni**	100.400	Zuidwolde (Netherlands)[b]	*Prunus laurocerasus* cv. Grüner Teppich	+	+	+
X. arboricola pv. *pruni**	100.439	Elburg (Netherlands)[b]	*Prunus laurocerasus* cv. Zabeliana	+	+	+
X. arboricola pv. *pruni**	CFBP 3894[PT]	New Zealand	*Prunus salicina*	+	+	+
X. arboricola pv. *pruni**	CFBP 5530	Italy	*Prunus persica*	+	+	+
X. arboricola pv. *pruni**	CFBP 5724	United States	*Prunus amygdalus*	+	+	+
X. arboricola pv. *pruni**	CITA 4	Aragón (Spain)	*Prunus persica* cv. Catherine	+	+	+
X. arboricola pv. *pruni**	CITA 9	Zaragoza (Spain)	*Prunus persica* cv. Merrill O`Henry	+	+	+
X. arboricola pv. *pruni**	CITA 11	Huesca (Spain)	*Prunus persica* cv. Richard Lady	+	+	+
X. arboricola pv. *pruni**	CITA 33	Teruel (Spain)	*Prunus amygdalus* cv. Guara	+	+	+
X. arboricola pv. *pruni**	CITA 46	Navarra (Spain)	*Prunus persica* cv. Summer Lady	+	+	+
X. arboricola pv. *pruni**	CITA 70	Zaragoza (Spain)	*Prunus persica* x *Prunus amygdalus*	+	+	+
X. arboricola pv. *pruni*	CITA 154	Zaragoza (Spain)	*Prunus amygdalus*	+	+	+
X. arboricola pv. *pruni**	IVIA 2626.1	Badajoz (Spain)	*Prunus salicina* cv. Fortuna	+	+	+
X. arboricola pv. *pruni*	IVIA 2626.3	Badajoz (Spain)	*Prunus salicina* cv. Fortuna	+	+	+
X. arboricola pv. *pruni*	IVIA 2626.6	Badajoz (Spain)	*Prunus salicina* cv. Fortuna	+	+	+
X. arboricola pv. *pruni*	IVIA 2626.7	Badajoz (Spain)	*Prunus salicina* cv. Fortuna	+	+	+
X. arboricola pv. *pruni*	IVIA 2647.1.3	Badajoz (Spain)	*Prunus salicina* cv. Larry Ann	+	+	+
X. arboricola pv. *pruni**	IVIA 2647.1-2	Badajoz (Spain)	*Prunus salicina* cv. Larry Ann	+	+	+
X. arboricola pv. *pruni*	IVIA 2647.1-13	Badajoz (Spain)	*Prunus salicina* cv. Larry Ann	+	+	+
X. arboricola pv. *pruni*	IVIA 2647.3-2	Badajoz (Spain)	*Prunus salicina* cv. Friar	+	+	+
X. arboricola pv. *pruni**	IVIA 2647.3-1	Badajoz (Spain)	*Prunus salicina* cv. Friar	+	+	+
X. arboricola pv. *pruni*	IVIA 2649.10	Badajoz (Spain)	*Prunus salicina* cv. Friar	+	+	+
X. arboricola pv. *pruni*	IVIA 2649.3	Badajoz (Spain)	*Prunus salicina* cv. Friar	+	+	+
X. arboricola pv. *pruni*	IVIA 2649.7	Badajoz (Spain)	*Prunus salicina* cv. Friar	+	+	+
X. arboricola pv. *pruni*	IVIA 2758.2	Badajoz (Spain)	*Prunus salicina*	+	+	+
X. arboricola pv. *pruni*	IVIA 2758.3	Badajoz (Spain)	*Prunus salicina*	+	+	+
X. arboricola pv. *pruni**	IVIA 2826.1	Valencia (Spain)	*Prunus salicina* cv. Anna Gold	+	+	+
X. arboricola pv. *pruni*	IVIA 2826.10	Valencia (Spain)	*Prunus persica* cv. Zephir	+	+	+
X. arboricola pv. *pruni*	IVIA 2826.11	Valencia (Spain)	*Prunus persica* cv. Zephir	+	+	+
X. arboricola pv. *pruni*	IVIA 2826.3	Valencia (Spain)	*Prunus salicina* cv. Anna Gold	+	+	+

X. arboricola pv. *pruni*	IVIA 2826.4	Valencia (Spain)	*Prunus salicina* cv. Anna Gold	+	+	+
X. arboricola pv. *pruni*	IVIA 2826.5	Valencia (Spain)	*Prunus salicina* cv. Anna Gold	+	+	+
X. arboricola pv. *pruni*	IVIA 2826.6	Valencia (Spain)	*Prunus salicina* cv. Anna Gold	+	+	+
X. arboricola pv. *pruni*	IVIA 2826.9	Valencia (Spain)	*Prunus persica* cv. Zephir	+	+	+
X. arboricola pv. *pruni**	IVIA 2832.10	Valencia (Spain)	*Prunus salicina* cv. Angeleno	+	+	+
X. arboricola pv. *pruni*	IVIA 2832.17	Valencia (Spain)	*Prunus salicina* cv. Larry Ann	+	+	+
X. arboricola pv. *pruni*	IVIA 2832.19	Valencia (Spain)	*Prunus salicina* cv. Larry Ann	+	+	+
X. arboricola pv. *pruni*	IVIA 2832.21	Valencia (Spain)	*Prunus persica* cv. Zephir	+	+	+
X. arboricola pv. *pruni*	IVIA 2832.24	Valencia (Spain)	*Prunus salicina* cv. Anna Gold	+	+	+
X. arboricola pv. *pruni*	IVIA 2832.26	Valencia (Spain)	*Prunus persica* cv. 58CC70	+	+	+
X. arboricola pv. *pruni*	IVIA 2832.30	Valencia (Spain)	*Prunus persica* x *P. davidiana* (Cadaman®)	+	+	+
X. arboricola pv. *pruni*	IVIA 2832.5	Valencia (Spain)	*Prunus persica* x *P. davidiana* (Cadaman®)	+	+	+
X. arboricola pv. *pruni**	IVIA 3161.2	Alicante (Spain)	*Prunus amygdalus* cv. Rumbeta	+	+	+
X. arboricola pv. *pruni**	IVIA 3162	Alicante (Spain)	*Prunus amygdalus* cv. Rumbeta	+	+	+
X. arboricola pv. *pruni*	IVIA 3177.1-6	Alicante (Spain)	*Prunus amygdalus* cv. Rumbeta	+	+	+
X. arboricola pv. *pruni*	IVIA 3177.3-4	Alicante (Spain)	*Prunus amygdalus* cv. Rumbeta	+	+	+
X. arboricola pv. *pruni*	IVIA 3177.3-8	Alicante (Spain)	*Prunus amygdalus* cv. Rumbeta	+	+	+
X. arboricola pv. *pruni*	IVIA 3181.3-1	Alicante (Spain)	*Prunus amygdalus* cv. Rumbeta	+	+	+
X. arboricola pv. *pruni*	IVIA 3181.3-3	Alicante (Spain)	*Prunus amygdalus* cv. Rumbeta	+	+	+
X. arboricola pv. *pruni**	IVIA 3487.1	Huesca (Spain)	*Prunus armeniaca*	+	+	+
X. arboricola pv. *pruni*	IVIA 4490.1	Teruel (Spain)	*Prunus amygdalus* cv. Rumbeta	+	+	+
X. arboricola pv. *pruni*	IVIA 4491.1	Huesca (Spain)	*Prunus amygdalus* cv. Rumbeta	+	+	+
X. arboricola pv. *pruni*	IVIA 4493	Huesca (Spain)	*Prunus amygdalus* cv. Rumbeta	+	+	+
Xap-look-a-like	CITA 14	Zaragoza (Spain)	*Prunus persica* cv. Luciana	+	-	-
Xap-look-a-like	CITA 42	Zaragoza (Spain)	*Prunus mahaleb* rootstock (Santa Lucía SL-64)	+	-	-
Xap-look-a-like*	CITA 44	Zaragoza (Spain)	*Prunus mahaleb* rootstock (Santa Lucía SL-64)	+	-	-
Xap-look-a-like	CITA 49	Zaragoza (Spain)	*Prunus* sp.	+	-	-
Xap-look-a-like	CITA 51	Zaragoza (Spain)	*Prunus amygdalus*	+	-	-
Xap-look-a-like	CITA 124	Badajoz (Spain)	*Prunus persica*	+	-	-
Xap-look-a-like	CITA 149	Guipúzcoa (Spain)	*Prunus laurocerasus*	+	-	-
X. arboricola pv. *corylina**	CFBP 1846	France	*Corylus avellana*	+	-	-
X. arboricola pv. *corylina**	IVIA 3978	Spain	*Corylus avellana*	+	-	-
X. arboricola pv. *corylina**	RIPF-X08	Poland	*Corylus avellana*	-	-	-
X. arboricola pv. *fragariae**	CFBP 6771[PT]	Italy	*Fragaria x ananassa*	-	-	-
X. arboricola pv. *juglandis**	IVIA 2113	Badajoz (Spain)	*Juglans regia*	-	-	-

X. arboricola pv. *juglandis**	CITA Xaj-2	Zaragoza (Spain)	*Juglans regia*	-	-	-
X. arboricola pv. *juglandis**	CITA Xaj-3	Zaragoza (Spain)	*Juglans regia*	-	-	-
X. arboricola pv. *juglandis**	CITA Xaj-4	Zaragoza (Spain)	*Juglans regia*	-	-	-
X. arboricola pv. *juglandis**	CITA Xaj-5	Zaragoza (Spain)	*Juglans regia*	-	-	-
X. arboricola pv. *populi**	CFBP 3123[PT]	Netherlands	*Populus x euroamericana* cv. Robusta	-	-	-
**Other pathogenic species**						
***Xanthomonas* spp.**						
X. campestris	CITA Xca-1	Teruel (Spain)	*Brasssica oleraceae*	-	-	-
X. campestris	CITA Xca-2	Tarragona (Spain)	*Brasssica oleraceae*	-	-	-
X. campestris	IVIA 2734.1	Spain	*Brassica mathis*	-	+	-
X. citri subsp. citri	306	Brazil	*Citrus sinensis*	+	+	-
X. citri subsp. *citri*	IVIA 2889-1	Argentina	*Citrus sinensis*	-	+	-
X. citri subsp. *citri*	IVIA 3026-1	Spain	*Citrus sinensis*	-	+	-
X. fuscans subsp. *fuscans*	NCPPB 381	Canada	*Phaseolus vulgaris*	-	+	-
X. fuscans subsp. *fuscans*	IVIA 151835DA	La Rioja (Spain)	*Phaseolus vulgaris*	-	+	-
X. hortorum pv. *pelargonii*	CITA Xp-2	Navarra (Spain)	*Pelargonium* sp.	-	+	-
X. hortorum pv. *pelargonii*	IVIA 1575.1	Spain	*Pelargonium peltatus*	-	-	-
X. vesicatoria	IVIA 3619-1	Spain	*Capsicum annum*	-	+	-
Agrobacterium						
A. tumefaciens	IVIA B1-360-1	NA	NA	-	-	NA
A. tumefaciens	CITA Agrob-9	Zaragoza (Spain)	*Prunus persica*	-	-	NA
Agrobacterium sp.	IVIA 1245-80	Spain	*Prunus persica* cv. Adafuel	-	-	NA
Agrobacterium sp.	IVIA 2304-10	Spain	*Prunus persica* cv. Red Candel	-	-	NA
Pantoea						
Pantoea sp.	IVIA 2261-1	Spain	*Olea europea* cv. Picual	-	-	NA
Pseudomonas						
P. syringae pv. *syringae*	IVIA 3514-2	Spain	*Citrus sinensis* cv. Valencia Late	-	-	NA
P. syringae pv. *syringae*	CITA Psy-3	Zaragoza (Spain)	*Prunus avium*	-	-	NA
P. syringae pv. *syringae*	CITA Psy-13	Zaragoza (Spain)	*Prunus amygdalus*	-	-	NA
**Commensal strains on *Prunus* spp.**						
Curtobacterium sp.	CITA EP-2.2	Teruel (Spain)	*Prunus amygdalus* cv. Guara	-	-	NA
Curtobacterium sp.	CITA EP-18.1	Teruel (Spain)	*Prunus amygdalus* cv. Guara	-	-	NA
Microbacterium sp.	CITA EP-16.1	Teruel (Spain)	*Prunus amygdalus* cv. Guara	-	-	NA
Pantoea sp.	CITA EP-14.1	Teruel (Spain)	*Prunus amygdalus* cv. Guara	-	-	NA
Pseudoclavibacter sp.	CITA EP-16.4	Teruel (Spain)	*Prunus amygdalus* cv. Guara	-	-	NA
Pseudomonas sp.	CITA EP-17.1	Teruel (Spain)	*Prunus amygdalus* cv. Guara	-	-	NA
Pseudomonas sp.	CITA 21/14-12.B8	Zaragoza (Spain)	*Prunus persica* cv.	-	-	NA

			Guayox 30			
Sphingomonas sp.	CITA EP-16.2	Teruel (Spain)	*Prunus amygdalus* cv. Guara	-	-	NA
Terrabacter sp.	CTA EP-16.6	Teruel (Spain)	*Prunus amygdalus* cv. Guara	-	-	NA

*Strains characterized previously as *Xanthomonas arboricola* and used in this study as control during the molecular characterization.

[PT]: Pathotype strain; NA: not available information; +: Positive PCR result; -: Negative PCR result.

[a]CFBP, Collection Française de Bactéries Phytopathogénes, Angers, France; CITA, Centro de Investigación y Tecnología Agroalimentaria de Aragón, Zaragoza, Spain; IVIA, Instituto Valenciano de Investigaciones Agrarias, Valencia, Spain; NCPPB, National Collection of Plant Pathogenic Bacteria, Sand Hutton, United Kingdom.[b]National Reference Laboratory, Plant Protection Service (Wageningen, NL).
